# Nanocellulose: Recent Advances Toward Biomedical Applications

**DOI:** 10.1002/smsc.202200076

**Published:** 2022-12-22

**Authors:** Xuan-Ran Ong, Adrielle Xianwen Chen, Ning Li, Yi Yan Yang, He-Kuan Luo

**Affiliations:** ^1^ Agency for Science, Technology and Research Institute of Sustainability for Chemicals, Energy and Environment 1 Pesek Road, Jurong Island Singapore 627833 Singapore; ^2^ Agency for Science, Technology and Research Institute of Bioengineering and Bioimaging 31 Biopolis Way Singapore 138669 Singapore

**Keywords:** antimicrobials, drug delivery, immobilization, implants, nanocellulose, tissue engineering, wound healing

## Abstract

Sustainable materials are key to the continual improvement of living standards on this planet with minimal environmental impacts. Nanocellulose combines the fascinating features of nanomaterials with favorable properties of the abundantly available cellulose biopolymer, which in recent years has gained much attention toward biomedical applications by virtue of its unique surface chemistry, remarkable physical features, and inherent biological attributes. Herein, the recent advances in nanocellulose‐based biomedical materials, with foci on biomolecule immobilization, drug delivery, cell culture and tissue engineering (TE), antimicrobial strategy, wound healing, and biomedical implants are summarized. Each topic is elaborated with representative examples to present the significance of nanocelluloses in their respective material design principles utilizing different sub‐types, including cellulose nanofibers (CNFs), cellulose nanocrystals (CNCs), and bacterial nanocellulose (BNC). The current state of large‐scale production of nanocellulose and accelerated development by artificial intelligence and machine learning are also briefly discussed, before ending with its future prospects and potential challenges.

## Graphical abstract

Recent advances in nanocellulose‐based biomedical materials are summarized, with emphasis on the sub‐types of nanocellulose (nanofibers, nanocrystals, bacterial nanocellulose) and their respective applications for biomolecule immobilization, drug delivery, cell culture and tissue engineering (TE), antimicrobial designs, wound healing, and biomedical implants. The large‐scale production of nanocellulose, its accelerated development by AI, future prospects, and challenges are also discussed.

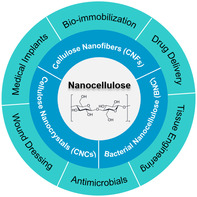

## 1 Introduction

With an estimated annual production of 10^11^ to 10^12^ tons and a negative carbon footprint, cellulose represents a readily accessible and environmentally benign material that holds great promise to support a green and sustainable future.^[^ ^1^ ^]^ For centuries, cellulose has been widely employed in the form of timber blocks and natural fibers as energy sources, building materials, and household textiles, which have all played important roles in improving living conditions and shaping civilization's progress on Earth.^[^ ^2^ ^]^ The first scientific discovery of cellulose was made in France in 1838, when Anselme Payen observed a fibrous solid residue after treatment of plant tissues with acids/ammonia and extraction using water and organic solvents. Its chemical formula was determined to be C_6_H_10_O_5_ by elemental analysis,^[^ ^3^ ^]^ though the polymeric structure of cellulose was not known to us until the 1920s.^[^ ^4^ ^]^ Since then, there have been continuous research efforts directed toward this naturally occurring and abundantly available biopolymer material, with a number of breakthroughs made on its dissolution,^[^ ^5^ ^]^ chemical modification,^[^ ^6^ ^]^ and wide‐ranging technological applications.^[^ ^7^ ^]^

**Figure 1:**
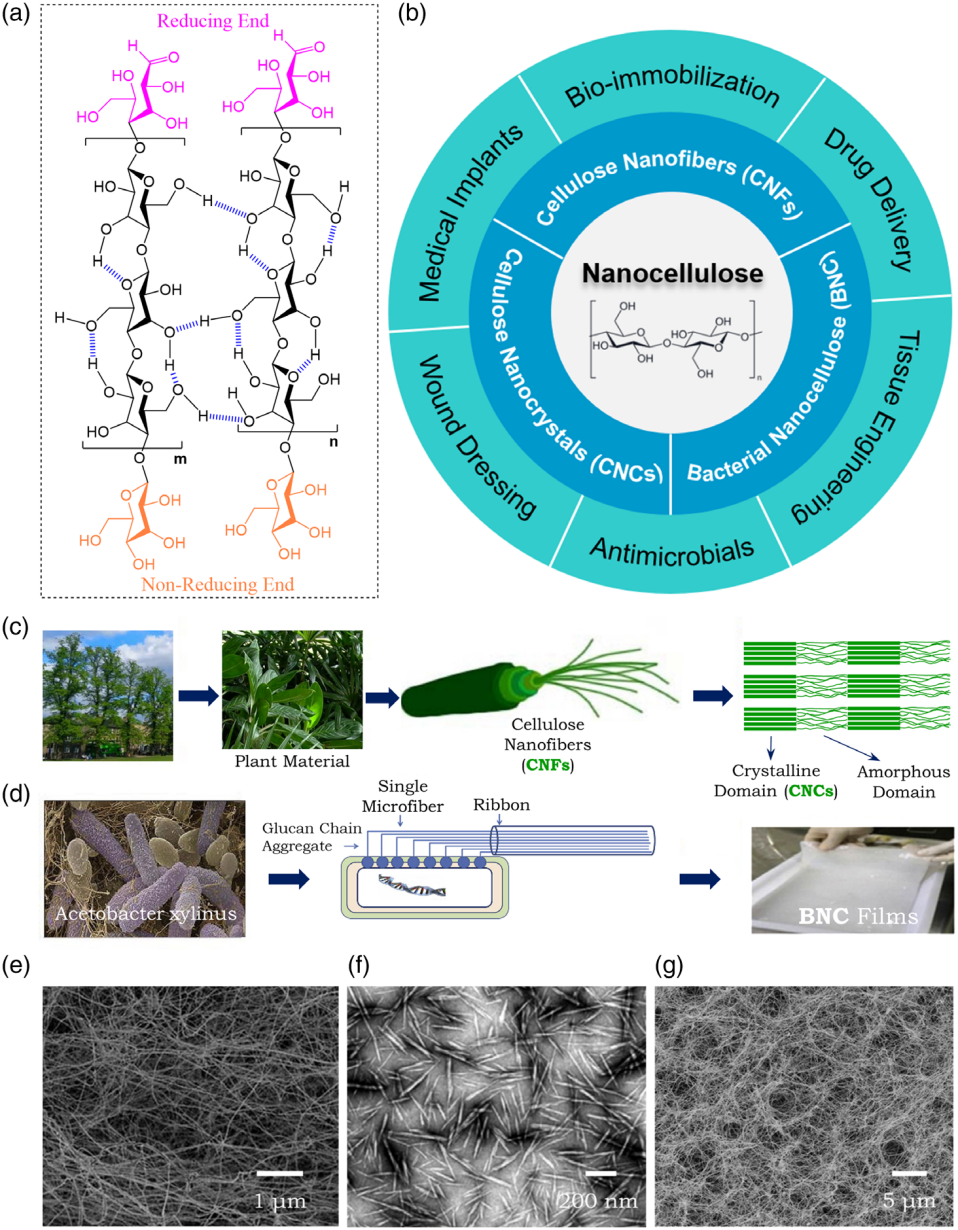
a) Illustration of the polymeric structure of cellulose, as well as its intra‐ and inter‐molecular hydrogen bonds. b) Schematic diagram presenting different types of nanocelluloses and their biomedical applications. c) The hierarchical structure of plant tissue and its derived nanocellulose. d) Scanning electron microscopy (SEM) image of the bacterial nanocellulose (BNC)‐producing *Acetobacter xylinus* and the BNC formation process. d) Reproduced with permission.^[^ ^10^ ^]^ Copyright 2014, Elsevier. e) SEM image of typical cellulose nanofibers (CNFs). Reproduced with permission.^[^ ^11^ ^]^ Copyright 2017, Royal Society of Chemistry. f) Transmission electron microscopy (TEM) image of typical CNCs. Reproduced with permission.^[^ ^12^ ^]^ Copyright 2008, Royal Society of Chemistry. g) SEM image of typical BNC. Reproduced with permission.^[^ ^13^ ^]^ Copyright 2017, Royal Society of Chemistry.

The general chemical structure of cellulose is a linear polymer comprising inter‐connected β‐1,4‐anhydro‐d‐glucopyranose units (AGUs), with every other AGU rotated by 180° in the plane to accommodate the preferred bond angles of the oxygen bridge in between (**Figure** **1**a). As a result, dimer cellobiose is usually seen as the repeating unit of cellulose. The unidirectional parallel orientation of the polymer chains leaves hemiacetal functionality on one end of the cellulose fibers (the reducing end, highlighted in pink, Figure 1a) and intact hydroxyl groups on the other (the non‐reducing end, highlighted in orange). Moreover, many hydrogen bonds can be formed between the ubiquitous hydroxyl groups on cellulose with vicinal oxygen centers, which is of critical importance in the formation of the fibrous morphology and the semicrystalline packing of cellulose, as well as its excellent mechanical strength.^[^ ^8^ ^]^ “Nanocellulose” refers to cellulosic extracts consisting of nanosized structures and components.^[^ ^9^ ^]^ Considering its sources of origin and production methods, nanocellulose can be further categorized into three sub‐types which include 1) cellulose nanofibers (CNFs)—also termed cellulose nanofibrils, nanofibrillated cellulose, or microfibrillated cellulose (Figure 1c); electrospun nanofibers of cellulose are also categorized into this group; 2) cellulose nanocrystals (CNCs)—likewise termed cellulose nanowhiskers, cellulose nanorods, or nanocrystalline cellulose (Figure 1c); 3) bacterial nanocellulose (BNC)—sometimes without the “nano” designation, or also named as microbial nanocellulose (Figure 1d).

CNFs consist of fibrous components with large aspect ratios. Being flexible in nature, most CNFs adopt a 3D porous morphology (Figure 1e) wherein fiber entanglement makes it impractical to experimentally determine the lengths of individual nanofibers. Therefore, only cross‐sectional dimensions of CNFs are documented in the literature, with generally agreed fiber diameters spanning 10–100 nm.^[^ ^14^ ^]^ CNFs are commonly obtained from plant‐based raw materials (e.g., wood, cotton, wheat straw, sugar beet, etc.) via 1) chemical (e.g., 2,2,6,6‐tetramethylpiperidine‐1‐oxyl (TEMPO)‐mediated oxidation,^[^ ^15^ ^]^ acid hydrolysis^[^ ^16^ ^]^) and/or enzymatic treatment in the wet state,^[^ ^17^ ^]^ or 2) top‐down mechanical defibrillation (e.g., high‐pressure homogenization, milling, high‐intensity ultrasonication, microfluidization, cryocrushing).^[^ ^18^ ^]^ These procedures ensure effective delamination of bulk cellulose and a complete conversion of raw cellulose from its large macro‐scale units to smaller nanofibers. For individual CNFs, both crystalline and amorphous domains are present in an alternating manner along its longitudinal direction (Figure 1c), with the former accounting for its elasticity and stiffness, and the latter for flexibility and plasticity.

CNCs are crystalline derivatives extracted by chemical‐induced disintegration such as sulfuric acid hydrolysis (or less commonly by oxidation) to selectively remove the amorphous domains in native cellulose. Upon hydrolysis, esterification between the inherent hydroxyl groups and extraneous sulfuric acid molecules produces sulfate esters on the CNC surface, making the resultant crystallites negatively charged and soluble in an aqueous environment.^[^ ^19^ ^]^ This feature makes CNCs compatible with physiological conditions and also improves their large‐scale processibility. The morphology of CNCs is generally a nano‐rod or nano‐needle, made of defect‐free, rigid cellulose crystallite (Figure 1f). Plant‐extracted CNCs are usually 5–30 nm in diameter and 100–500 nm in length, whereas those from other sources such as tunicate and algae could be up to several microns long.^[^ ^20^ ^]^

In contrast to CNFs and CNCs, the production of BNC is a bottom‐up process, in which the starting glucose monomers are assembled into polymer chains within appropriate bacterial species (e.g., *Acetobacter xylinum*) (Figure 1d). The polymer chains then pass through the pores in the bacteria cell wall and aggregate into nanofibers of about 20–100 nm in diameter, before further forming web‐like network structures in the cultivation medium (Figure 1g). Cultivation methods include: 1) static cultures—which form a gelatinous membrane of BNC nanofibers at the culture media air interface; 2) agitated cultures—which produce irregular or pellet‐like BNC masses; 3) culturing in bioreactors.^[^ ^21^ ^]^ BNC is usually of higher chemical purity than CNFs and CNCs, hence additional steps to remove undesired impurities like lignin and pectin commonly found in plant‐derived nanocellulose are not necessary.^[^ ^22^ ^]^ The properties and yield of synthesized BNC are dependent on the type of bacterial strains used, as well as culture conditions (e.g., pH, temperature, oxygen, and nutrition levels).^[^ ^23^ ^]^ BNCs exhibit a higher degree of polymerization and crystallinity than CNFs while possessing high fibrosity, remarkable water absorption capabilities, and tunable water content, all of which present favorable attributes for use in biological settings.

By virtue of their intriguing properties, biological origins, and natural abundance, the use of nanocellulose in biomedical applications constitutes an important and fast‐paced research domain to which numerous efforts have been devoted in recent years. This initiative is also in line with the rising demand for sustainable materials to advance healthcare and biomedical technologies targeted toward improving well‐being and prolonging the human lifespan. Many excellent reviews have been published in this field, with a majority being focused on BNC and its derivatives.^[^ ^21^, ^23^, ^24^ ^]^ In the current article, we present the recent advances of all three sub‐types of nanocellulose (i.e., CNFs, CNCs, and BNC) and their biomedical applications. Covered themes include biomolecule immobilization, programmable drug delivery, cell culture and tissue engineering (TE), antimicrobial nanomaterials, wound dressing and healing, and biomedical implants (Figure 1b). In each topic, the role of nanocellulose is elaborated using representative examples with emphasis on their material/device design principles utilizing different nanocelluloses. Current views, prospects, and challenges faced in each area are also reviewed with suggestions made for future progress.

## 2 Biomedical Applications of Nanocellulose

### 2.1 Biomolecule Immobilization

After isolation from their native environments, many biomolecules suffer from low stability and poor recyclability for in vitro applications. Immobilization of such biomolecules in a scaffold matrix is a promising approach to overcome these drawbacks. Ideally, the matrix ought to be biocompatible and easily programmable with enhanced loading efficiency and system stability for long‐term operation and storage. Possessing high mechanical strength and excellent biocompatibility, nanocellulose represents a prominent immobilization material of such kind.^[^ ^25^ ^]^ Nonetheless, as the inherent hydroxyl groups of nanocelluloses interact only weakly with many biomolecules,[^24^, ^26^] they usually require surface modification by amine, epoxy, aldehyde, or carboxyl groups to ensure adequate binding of proteins,^[^ ^27^ ^]^ DNA molecules^[^ ^28^ ^]^ and peptides.^[^ ^29^ ^]^

**Figure 2:**
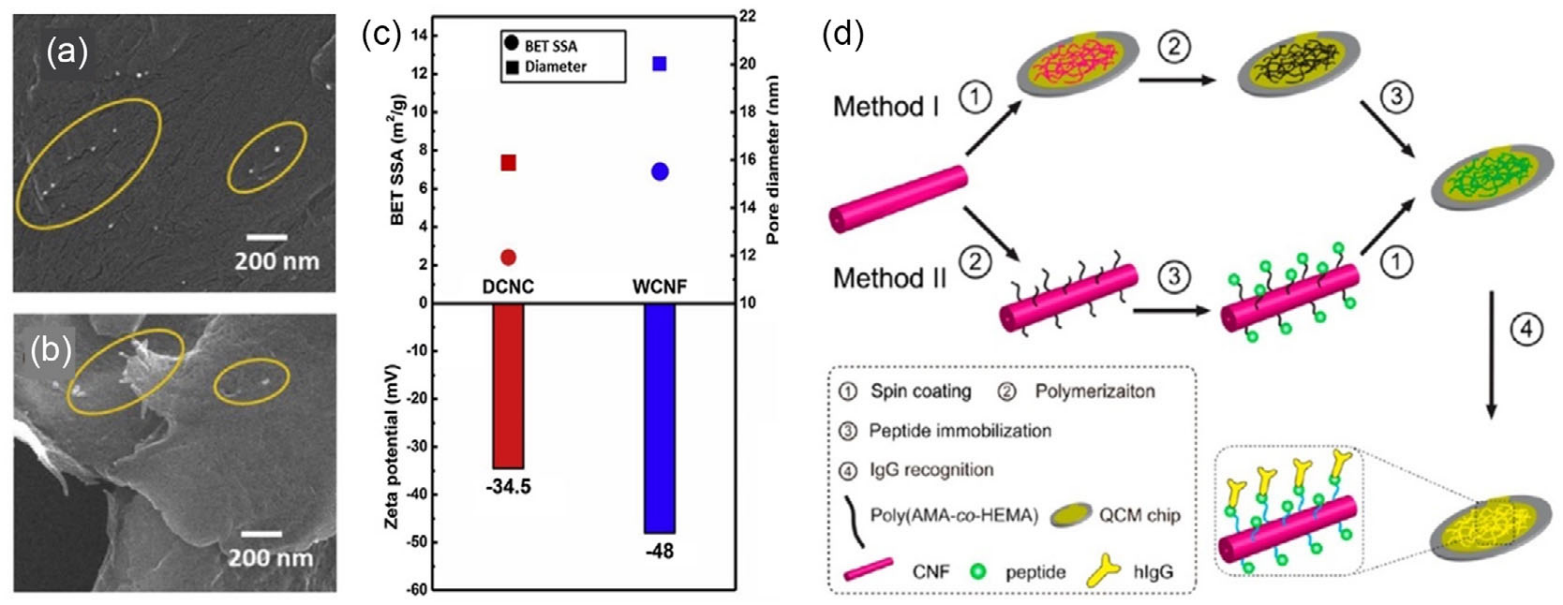
a,b) SEM images of deep eutectic solvent‐fabricated cotton CNCs (DCNC) (a), and TEMPO‐oxidized wood CNFs (WCNF) (b), with immobilized human neutrophil elastase (HNE) peptides (circled in yellow). c) Top graph: average specific surface area (SSA) (square) and pore sizes (circle) of DCNCs (in red) and WCNFs (in blue); bottom graph: surface charge of DCNC and WCNF. a–c) Reproduced with permission.^[^ ^32^ ^]^ Copyright 2019, Elsevier. d) Preparation procedures of the spacer‐conjugated peptide‐CNF films for hIgG detection. Reproduced with permission.^[^ ^33^ ^]^ Copyright 2013, American Chemical Society.

Many early studies have demonstrated significant improvements in bioactivity, thermal and pH stability, and material recyclability of nanocellulose‐immobilized biomolecules as compared to their free‐standing counterparts,[^27^, ^30^] whereas recent investigation sought to surpass these statistical ceilings while generating novelties in the application. Nanocellulose‐based biosensors constitute an emerging focus, with representative works including disposable glucose sensors assembled via glucose oxidase immobilization in a CNF cryogel composite, and subsequently spray‐deposited to pattern a microfluidic channel for enhanced colorimetric glucose detection.^[^ ^31^ ^]^ Interestingly, results from a comparison study between wood‐CNFs and cotton‐CNCs for the immobilization of a human neutrophil elastase (HNE) peptide revealed a higher immobilization effectiveness by the CNCs (**Figure** **2**a,b).^[^ ^32^ ^]^ These results were in spite of the CNF matrix's larger surface area and pore sizes (Figure 2c), but could be explained by its TEMPO‐oxidation synthesis method, which induced more carboxylic groups and negative charges, causing surface hindrance. Nonetheless, both materials showed good sensitivities in HNE detection, presenting the potential of nanocellulose and its immobilization strategies for fabricating cheap and compact biosensors. In addition, cases of complex conjugation chemistry may call for creative methods; copolymer‐grafted CNF films for short peptide conjugation were demonstrated for application in human immunoglobulin G (hIgG) detection (Figure 2d).^[^ ^33^ ^]^ The pre‐grafted copolymers not only act as spacers, but also support peptide immobilization for subsequent hIgG binding. Similar spacer‐conjugate strategies are particularly useful when specific target biomolecules need to be anchored in cases where conventional surface modification is inadequate or too complicated to achieve.^[^ ^34^ ^]^

Shape‐tailored nanocellulose immobilization matrices, such as microspheres, complement the inclusion of adventive functionalities, e.g., magnetic responsiveness for easy material manipulation and recovery.^[^ ^35^ ^]^ Additionally, biomolecule immobilization with assistance from metal nanoparticles can further aid in its bioactivity preservation.^[^ ^36^ ^]^ For example, acidic‐ and alkaline‐treated CNCs bonded to iron oxide nanoparticles as a magnetic immobilization material for β‐galactosidase enzymes showed improved thermal stabilities and high catalytic property retention rates.^[^ ^37^ ^]^ Both treated CNCs were capable of more than 30 reuse cycles while maintaining a high percentage of the enzyme's bioactivity—a significant improvement over previous studies.^[^ ^30^ ^]^ Follow‐up research developed sustainable single‐step immobilization methods using recombinant β‐galactosidase with the cellulose‐binding domain (CBD), producing nanocellulose composites with the reusability of up to 40 cycles while retaining 53 to 64% of its lactose hydrolysis capacity.^[^ ^38^ ^]^

Notably, biomolecule immobilization on CNCs can be accomplished in a site‐specific manner by leveraging on their anisotropic needle‐like nano morphology. The feasibility of attaching β‐casein on CNC crystallite tips was established by employing a click reaction between the azide‐carrying CNC‐reducing end and the acetylene‐carrying β‐casein micelles, which facilitates the formation of conjugated nanoparticles in a mushroom‐like configuration.^[^ ^39^ ^]^ Similar strategies have enabled the immobilization of labeled DNA and peptide sequences on CNCs as bio‐probes, which are subsequently employed for the identification and recognition of target biomolecules, or as platforms for immunoassays, diagnostics, and gene delivery applications.^[^ ^40^ ^]^

An advantage of BNC immobilization matrices is in their tailorable fabrication of desired architectures. In dimension‐controlled spherical alginate/BNC nanocomposite beads constructed via entrapment of *Gluconacetobacter xylinus* in barium alginate hydrogels, the incorporation of BNC improved the surface area, crystallinity, and water vapor sorption capacity of the nanocomposite beads.^[^ ^41^ ^]^ Moreover, specific bioactivity of immobilized lipase was up to 3.8 times higher on the beads than that on pure cellulose of similar morphology, demonstrating synergistic effects of the different composite components on biomolecule immobilization. BNC can also be tailored for use in wearable sensors or electrodes as biomolecule immobilization substrates. Gomes et al. immobilized lactate oxidase on a sodium m‐periodate‐oxidized BNC substrate as part of a lactate biosensor which exhibited excellent mechanical resistance and biocompatibility.^[^ ^42^ ^]^ Its successful wide‐range detection of lactate concentrations from 1.0 to 24.0 mmol L^−1^ demonstrates the potential of bio‐immobilized BNCs as promising materials for flexible and wearable sensors.

Recent rapid advances in the adoption of artificial intelligence for bioinformatics and bioimaging have enabled efficient and large‐scale analyses of data samples generated from biosensors. An example is demonstrated in lectin‐conjugated BNC nanocrystals used as bacteria binding and detection assays for label‐free surface‐enhanced Raman spectroscopy (SERS) biosensors.^[^ ^43^ ^]^ An optimization‐based machine learning technique, support vector machine (SVM), was employed as a binary classifier to analyze the large SERS dataset generated by 19 bacterial strains, which successfully differentiated between the strains with a high overall accuracy of 87.7%. In addition, machine learning algorithms and computer simulations are increasingly adopted for automating protocols for material fabrication and optimization, characterization, experimental result analyses, and are undoubtedly an upcoming research direction in the production and biomedical applications of nanocellulose and beyond.

**Figure 3:**
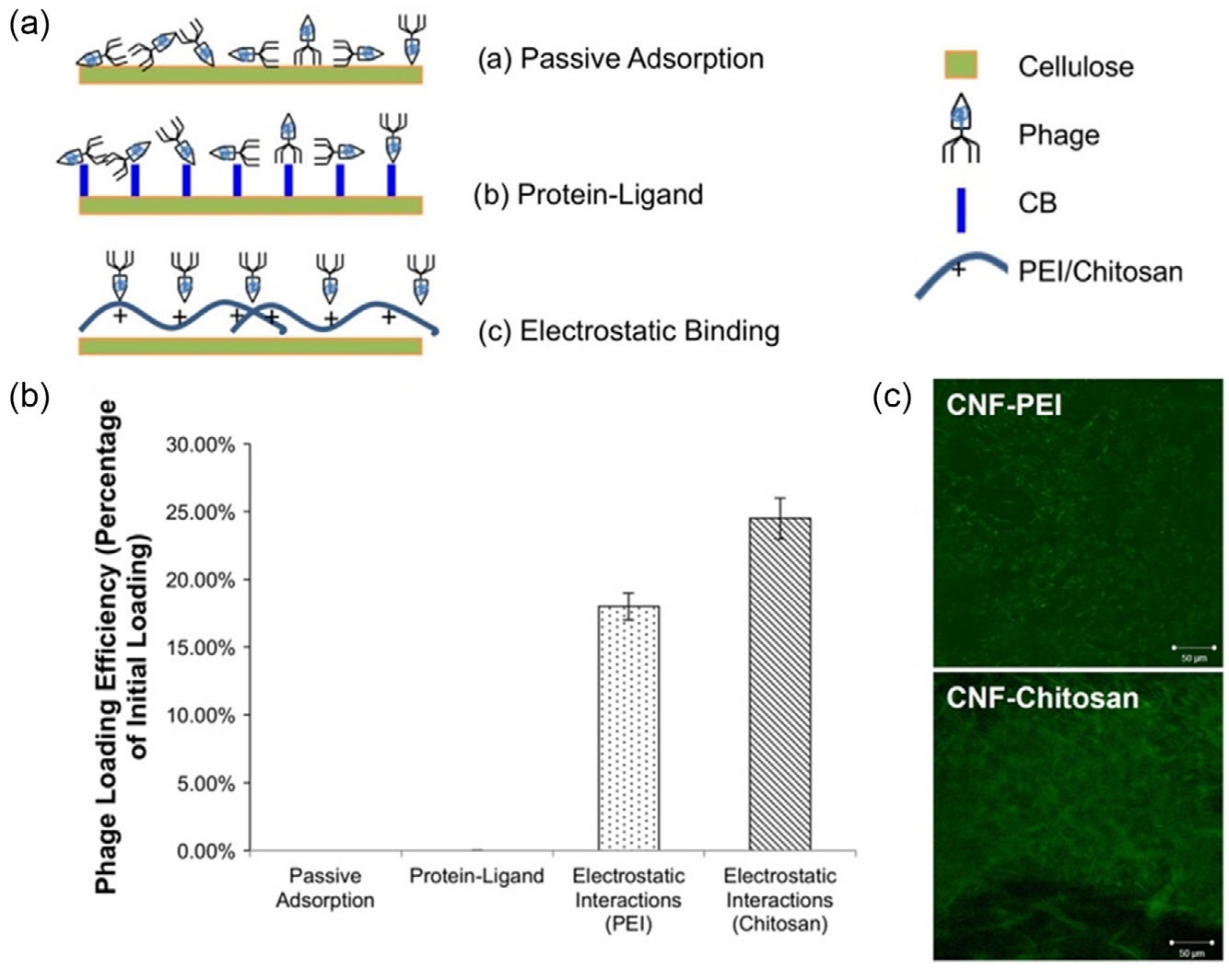
a) Illustration of physical immobilization methods—passive adsorption, protein–ligand binding, and electrostatic interactions. CB represents the Cibacron Blue F3GA dye ligand. b) Phage immobilizing efficiency normalized to its initial loading. Non–specific adsorption and protein–ligand binding yielded insignificant immobilization at <0.001% of initial phage loading. Electrostatically immobilized phages yielded approximately 16% and 24% loading on CNF‐PEI and CNF‐Chitosan respectively. c) Confocal images of fluorescently labeled T7 bacteriophages immobilized on CNF‐PEI and CNF‐Chitosan show a random and uniform phage distribution. a–c) Reproduced with permission.^[^ ^48^ ^]^ Copyright 2017, Springer Nature.

In avoidance of direct chemical modifications of biomolecules to preserve their activity, immobilization via physical adsorption is preferable and is especially suitable on porous CNF and BNC matrices.[^28^, ^44^] To enhance immobilization efficiency, prior modifications to the nanocellulose matrix (e.g., oxidation,^[^ ^45^ ^]^ copolymerization,^[^ ^46^ ^]^ biopolymer conjugation^[^ ^47^ ^]^) are oftentimes performed. The use of different physical adsorption methods can have varying effects on the immobilization efficiency, distribution on the nanocellulose substrate surface, and subsequent biomolecule release. Immobilization of T7 bacteriophages on electrospun CNFs was investigated via different physical methods, which found electrostatic binding as the most optimal approach over non‐specific adsorption and protein‐ligand binding (**Figure** **3**a,b).^[^ ^48^ ^]^ Electrostatic interactions between positively charged poly(ethylenenimine) (PEI)‐ and chitosan‐modified CNFs and the negatively charged phages achieved a uniform phage distribution (Figure 3c), and it was further demonstrated that fiber disintegration is the main mechanism for phage release. Hence, optimization of physical adsorption methods and their parameters has the potential for improving the applicability of biomolecule immobilized matrices to different uses.

It is worth additional notice that significant differences are observed between bacteria‐derived and plant‐derived nanocellulose in their biomolecule adsorption capacity. For instance, the protein adsorption capacity of phosphorylated BNC was found to be much higher than that of similarly phosphorylated CNFs.^[^ ^49^ ^]^ Similarly, quaternary ammonium BNC exhibited greater adsorption for hemoglobin but lower for thymol blue when compared to its CNF counterpart.^[^ ^50^ ^]^ Moreover, BNC produced by different bacteria strains also demonstrated varying adsorption towards laccase.^[^ ^51^ ^]^ These complexities could be ascribed to the different structural morphologies and surface chemistry of bacteria‐ and plant‐derived nanocellulose which necessitates further investigation.

### 2.2 Programmable Drug Delivery

Modern pharmaceuticals mostly come in the form of small chemical and biological molecules, which are susceptible to disintegration or degradation before reaching their physiological targets. Therefore, advanced drug delivery systems capable of protecting and controllably releasing drug components are desired for academic communities and industrial sectors. The intrinsic hydrophilicity of nanocellulose makes it merely capable of bonding with water‐soluble drugs such as doxorubicin and tetracycline,^[24d]^ whereas surface modification is required for binding with hydrophobic molecules.[^24^, ^52^] The following subsections outline nanocellulose‐assisted drug delivery via oral, transdermal, and injection administration routes.

#### 2.2.1 Oral Administration

Orally administered drugs are usually in tablet form comprising pharmaceutically active ingredients and a carrier matrix excipient. Direct compression is perhaps the most convenient production technique for tablets, whereby excipient materials must be readily compactable upon the incorporation of the pharmaceutical ingredients and should preserve the contained drugs for controllable periods against diverse physiological environments.^[^ ^53^ ^]^

**Figure 4:**
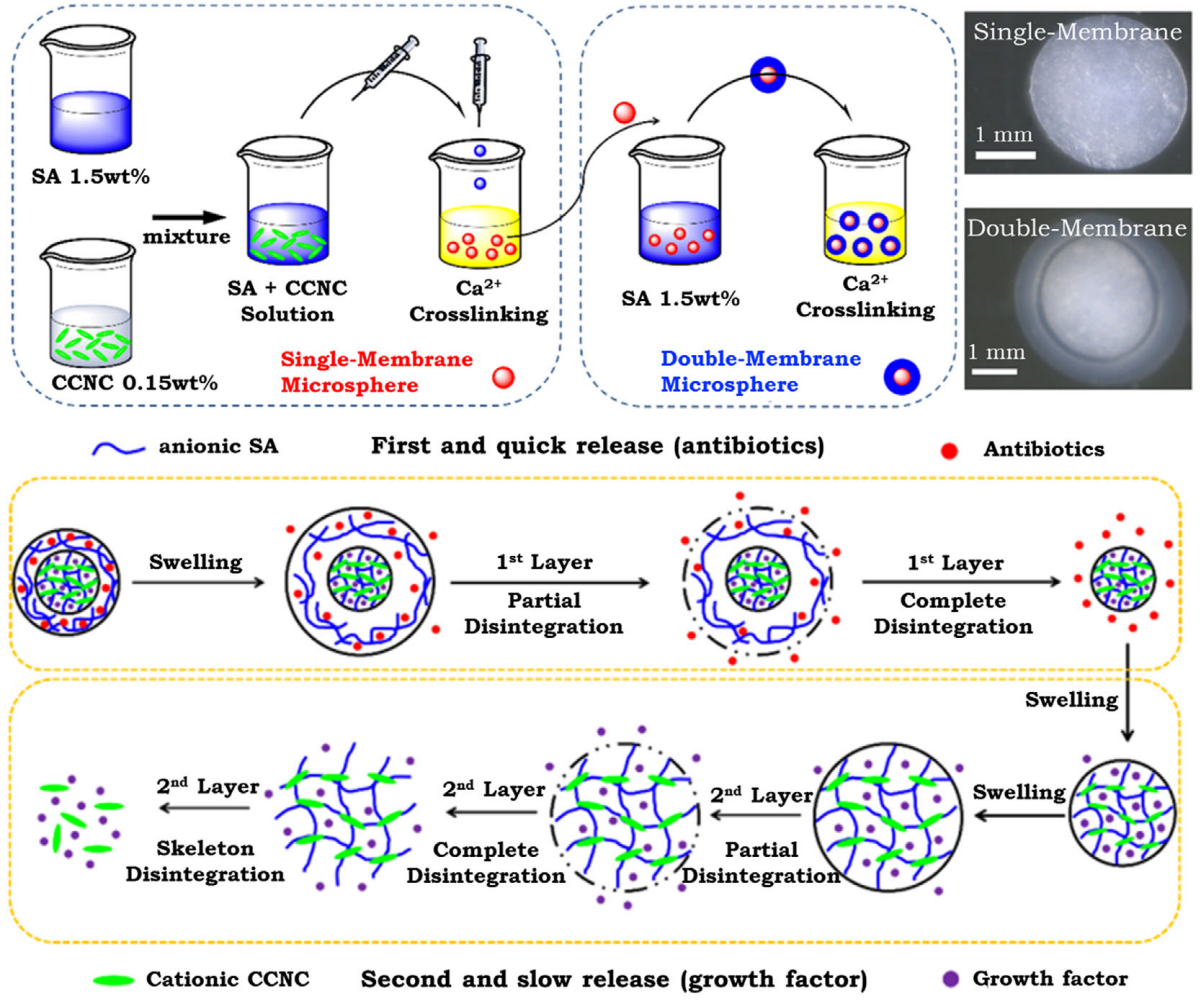
Top: Fabrication procedure of the single‐ and dual‐layer microsphere hydrogels, with their micro‐scale morphologies being shown on the right. Bottom: Proposed drug co‐release mechanism of the dual‐layer microsphere hydrogel. SA denotes sodium alginate, CCNC denotes chemically modified CNCs. Reproduced with permission.^[^ ^57^ ^]^ Copyright 2016, American Chemical Society.

Plant‐derived CNCs are a popular form of nanocellulose as an excipient due to their marked mechanical strength. They are commonly incorporated as additives,^[^ ^54^ ^]^ stabilizers,^[^ ^55^ ^]^ and coating materials^[^ ^56^ ^]^ in tablet matrices to improve material properties and to tune the drug release profiles. Notably, emerging classes of dual‐layered drug delivery systems can achieve customized co‐delivery, as illustrated by Lin et al.'s CNC/alginate double‐membrane hydrogel (**Figure** **4**, top panel).^[^ ^57^ ^]^ Drugs contained in the outer membrane could be rapidly discharged in appropriate media, whereas that in the inner layer exhibited slower release over an extended period (bottom panel). Such dual‐layer designs are particularly useful for the stepwise release of multiple drugs in a synergistic manner, providing a potential solution to drug compatibility and resistance issues.

Pristine CNFs are less commonly used for tablet production despite their notable performance in programmed drug release,^[^ ^58^ ^]^ due to pelleting difficulties. Nonetheless, CNF‐derived aerogels and hydrogels have been widely employed in state‐of‐the‐art pelleting systems, where the tablet disintegration rate and drug release profiles could be controlled by tuning gel compositions and surface chemistry via approaches such as microparticle inclusion,^[^ ^59^ ^]^ excipient layering,^[^ ^60^ ^]^ and crosslinking.^[^ ^61^ ^]^ Additionally, the rapidly developing 3D printing technologies offer advanced customizable drug fabrication options, with a recent example featuring sodium alginate‐CNF bio‐inks suitable for loading of both hydrophilic and hydrophobic drugs.^[^ ^62^ ^]^ These bio‐inks exhibited high printability into virtually any desired pharmaceutical conformation, as well as controllable drug release patterns by varying its post‐production processes. Temperature‐,^[^ ^63^ ^]^ magnetic‐,^[^ ^64^ ^]^ and pH‐responsiveness^[^ ^65^ ^]^ can also be introduced with ease in these CNF‐derived gels, allowing condition‐dependent drug release into target physiological areas within the body.

BNC's capability of inheriting its bacteria cultivation vessel shape presents the viability of producing hollow capsules for drug storage and release uses, and such BNC capsules in centi‐ and micro‐scale with controllable permeability and dissolution rate provide a green alternative to current capsule materials.^[^ ^66^ ^]^ Additionally, drug loading into BNC hydrogels via physical adsorption is a common encapsulation method, where the fine network in BNC matrices provides important characteristics for sustained drug release. Interesting works include the loading of poorly soluble compounds such as berberine hydrochloride for gastrointestinal infection treatments,^[^ ^67^ ^]^ achieved by forming an inclusion complex with cyclodextrins. The outer surface hydrophilicity of the complex promotes drug loading into BNC matrices, overcoming the hydrophobicity limitations of these drugs. Spray‐drying of BNC fibers with matrix formers such as mannitol into microsystems for drug loading is also a novel technique that can be further investigated.^[^ ^68^ ^]^

#### 2.2.2 Transdermal Administration

Transdermal drug delivery involves the therapeutic conveyance of small molecules through the skin, bypassing the physiological conditions in the gastrointestinal tract faced by their orally administered counterparts. Nanocellulose is commonly tailored into 2D films or membranes for incorporation of pharmaceutical ingredients including caffeine,^[^ ^69^ ^]^ crocin,^[^ ^70^ ^]^ curcumin,^[^ ^71^ ^]^ as well as various non‐steroidal anti‐inflammatory drugs (NSAIDs).^[^ ^72^ ^]^ Among these, BNC membranes are prominently employed as they can adhere firmly onto irregular skin surfaces, absorb skin exudates, and even be modified with skincare ingredients for added soothing benefits.^[^ ^73^ ^]^ A common consensus in transdermal mechanisms established the dependency of delivery efficiency on intrinsic drug molecular properties, wherein drug loading and release mechanisms involve an interplay between the carrier matrix, drug molecules, and the surrounding environment.

The emerging focus on methods to improve the loading of hydrophobic drugs has driven much progress in overcoming the inherent limitation of the hydrophilic pristine nanocellulose. Loading of hydrophobic and lipophilic compounds into nanoemulsions with lipidic carriers was successfully demonstrated using the coenzyme Q10 as a model drug.^[^ ^74^ ^]^ The nanoemulsions were then adsorbed onto BNC for dermal application without significant alterations to BNC's favorable characteristics. Nanocellulose can also be directly incorporated into drug‐loaded microemulsions, as demonstrated by Zainuddin et al.'s cetyltrimethylammonium bromide‐modified CNC microemulsions which improved skin retention and topical delivery of curcumin.^[71a]^ More recently, CNF composite hydrogels were conjugated with metal–organic frameworks for hydrophobic drug delivery using curcumin as a model.^[71b]^ Polydopamine was first polymerized on the CNF hydrogel, followed by in situ growth of zeolitic imidazolate frameworks (ZIF)‐8 nanoparticles and curcumin encapsulation. Exhibiting high drug loading ratios and tunable release periods of up to 4.5 wt% and 107 h, respectively, this innovative composite hydrogel presents a novel solution for hydrophobic drug delivery which can be further explored for its efficacy in the various drug administration routes.

#### 2.2.3 Injection Administration

**Figure 5:**
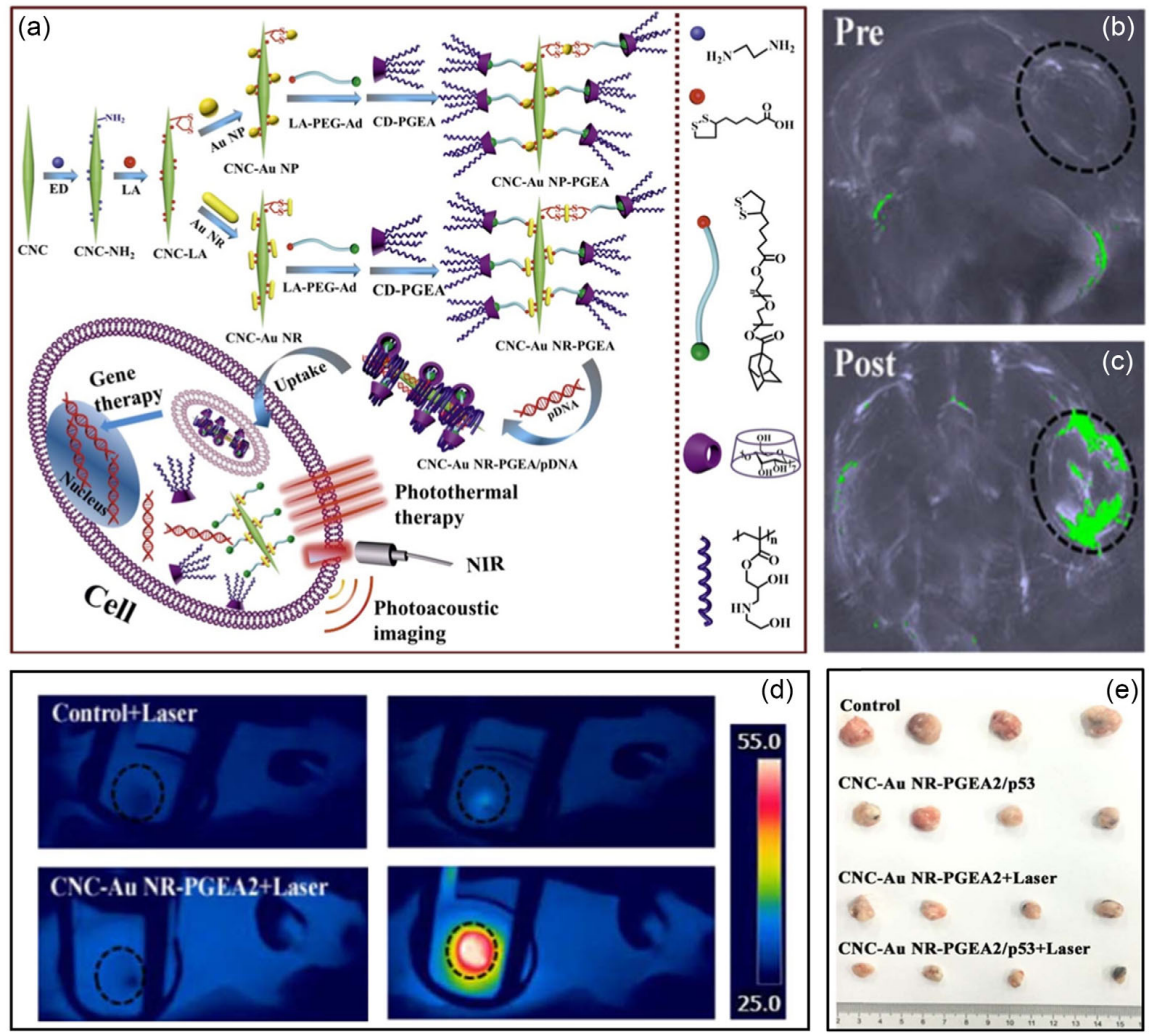
a) Preparation scheme of the CNC‐Au‐PGEA nanostructures, and their application in gene delivery and photoacoustic imaging. CD‐PGEA represents a molecular gene carrier. b,c) Photoacoustic images of mice before (b) and after (c) intratumoral injection of CNC‐AuNR‐PGEA. Tumor regions are highlighted in circles. d) Infrared (808 nm, 2.0 W cm^−2^) thermal images of a mouse bearing a C6 tumor after injection of PBS (control) and CNC‐AuNR‐PGEA. e) Digital photograph of tumors after the various treatments for 2 weeks. a–e) Reproduced with permission.^[^ ^79^ ^]^ Copyright 2017, Elsevier.

Local delivery systems involving direct drug release into or near target sites are often achieved via subcutaneous injection. To this end, the nanoparticulate CNCs are especially suitable for use as drug carriers in injectable gels which go into the blood circulation to reach their designated physiological target.^[^ ^75^ ^]^ Injectable CNC hydrogels produced by salt‐induced charge screening were successfully modeled for controlled drug release of doxorubicin, tetracycline, and the bovine serum albumin protein.^[^ ^76^ ^]^ This study provided important benchmarks to the characterization of injectable CNC hydrogel for drug delivery purposes, especially in: 1) its self‐healing and recovery of original matrix structures after injection; 2) drug release kinetics that are largely affected by intrinsic drug properties, drug–CNC interactions, and can be further modulated by incorporation of other biocompatible additives; 3) pH‐responsiveness. Other stimuli‐responsive nanocellulose materials for smart delivery systems are achieved via various polymer grafting strategies, exemplified in the covalent grafting of thermosensitive Jeffamine polyetheramine M2005 chains onto TEMPO‐oxidized CNCs.^[^ ^77^ ^]^ A potential application of stimuli‐responsive drug delivery is in cancer therapy via tumor embolization. Injectable, thermo‐responsive gels that undergo temperature‐induced phase transitions upon reaching target tumors were demonstrated by grafting poly(*N*‐isopropylacrylamide) on BNC nanocrystals.^[^ ^78^ ^]^ After injection, its phase transition into a stable gel at temperatures above 34.3 °C presents anticancer potential in blocking vascular exchange at tumor sites. Similarly, gene delivery of plasmid p53 for cancer therapy was achieved by thiol‐modified CNCs loaded with gold nanoparticles/nanorods and immobilized with the hydroxyl‐rich gene carrier CD‐PGEA (**Figure** **5**a).^[^ ^79^ ^]^ Notably, this CNC‐Au‐PGEA nanostructure combines gene transfection with photothermal ablation for an enhanced antitumor effect, which was validated by an in vivo rat glioma model (Figure 5b–e).

CNFs could also be used as injectable hydrogel matrices, with an example demonstrating TEMPO‐oxidized, Ca^2+^‐crosslinked CNF hydrogels modeled with ibuprofen for drug release.^[^ ^80^ ^]^ Its physiochemical and drug diffusion properties could be tuned by varying the amount of crosslinking cations and by drug complexion with carriers such as β‐cyclodextrin, respectively. Another example in cancer therapy demonstrated by a BNC anticancer drug carrier shows potential for treating peritoneally disseminated gastric cancers via intraperitoneal injection.^[^ ^81^ ^]^ Follow‐up research by Ando et al. validated this BNC carrier using doxorubicin as a model drug.^[^ ^82^ ^]^ The BNC/doxorubicin composite improved tumor suppression and reduced side effects of weight loss, as compared to the free drug itself.

### 2.3 Tissue Engineering and Regenerative Medicine

Tissue engineering (TE) combines biomaterials, cells, and signaling molecules in creating microenvironments with native tissue characteristics for regenerative treatment. Providing an in vitro simulation model of biological tissues, such TE constructs are also useful for disease modeling or as animal alternatives for drug testing.^[^ ^83^ ^]^ Due to its excellent mechanical strength, physicochemical properties, and biocompatibility, nanocellulose has garnered extensive interest as a natural scaffolding biomaterial for the recapitulation of native tissue environments.^[^ ^84^ ^]^ The fibrous morphology of nanocellulose (i.e., CNFs and BNC) is an additional advantage, as the fiber strands can act as nutrition transport channels for cells embedded deep inside the matrix.^[^ ^85^ ^]^ On the contrary, CNCs are usually employed as additives in matrices with other materials or functionalized into hydrogel forms for TE use, largely ascribed to their discrete nanoparticulate morphology.

The present‐day hot topic—3D bioprinting—undoubtedly extends into the field of cell and tissue culture, providing options for bottom‐up printed scaffolds and matrices using nanocellulose (see ref. [86] for selected reviews). Nanocellulose‐based hydrogels are a viable bio‐ink choice which possesses the required shear‐thinning properties and ability to form an extracellular matrix (ECM)‐like a 3D microenvironment.^[^ ^87^ ^]^ However, to ensure post‐print shape fidelity, incorporation of crosslinking agents (e.g., sodium alginate) is oftentimes required,^[^ ^88^ ^]^ and such crosslinking agents may further enable other useful features like mechanical property tuning and after‐use degradation.^[^ ^89^ ^]^

In the recapitulation of different tissue functions, TE scaffolds need to mimic the tissue‐specific native ECM and provide topographical and biochemical cues to direct cell growth and proliferation. In the following subsections, the use of nanocellulose and its unique considerations in cartilage, bone and neural cell culture and TE are elaborated.

#### 2.3.1 Cartilage TE

Articular cartilage defects resulting from joint injuries or degenerative diseases are the most widespread form of cartilage damage. For cartilage TE, nanocellulose has been shown to promote chondrogenesis and is often paired with hyaluronic acid and/or alginate to better simulate the native tissue matrix.^[^ ^90^ ^]^ Composite hydrogels based on CNFs, CNCs, and a blend of both—all with sodium alginate—were fabricated to investigate the effects of crosslinker concentration and sterilization method on the hydrogel properties.^[^ ^88^ ^]^ CNC‐based hydrogels were the most affected by increases in the CaCl_2_ concentration as a crosslinker and formed stiffer hydrogels, while the CNF‐ and blend‐based hydrogels showed properties mostly independent of crosslinker concentration. Cell viability for cartilage TE was assessed using human nasal‐septal chondrocytes which differentiated and proliferated well in the matrix. Interestingly, these nanocellulose hydrogels possess porosities and pore sizes that differ from typical values reported in other hydrogels for cartilage TE, thus possibly providing a broader perspective and base for investigating divergences from the “conventional” chondrogenic environment.

CNCs can also be used as nanofillers and/or crosslinkers in injectable chitosan/pectin hydrogels to provide mechanical reinforcement.^[^ ^91^ ^]^ By increasing the amount of CNC incorporated, hydrogels were formed with denser networks, smaller pore sizes, greater stiffness, and lower equilibrium swelling and degradability. Notably, scaffolds with a multilayered matrix design could improve the regeneration of deep osteochondral defects which extend into the subchondral bone. An exemplary work complexed two BNC‐based scaffold layers, BNC‐glycosaminoglycans and BNC‐hydroxyapatite, to mimic cartilage and bone, respectively.^[^ ^92^ ^]^ In vitro cell viability studies confirmed the chondro‐ and osteo‐inductive abilities of the respective modified BNC scaffolds. In vivo implantation of the combined acellular bilayer scaffold in a rat osteochondral defect model demonstrated accelerated and concurrent regeneration of both cartilage and bone tissues while exhibiting no observed immunoreactions. This premier study demonstrated the desirable degradation and bio‐resorption properties conferred by BNC and presents its modification flexibility for engineering different tissue types, paving the future for multilayered, nanocellulose‐based scaffold designs targeted at defects involving multiple tissue types.

**Figure 6:**
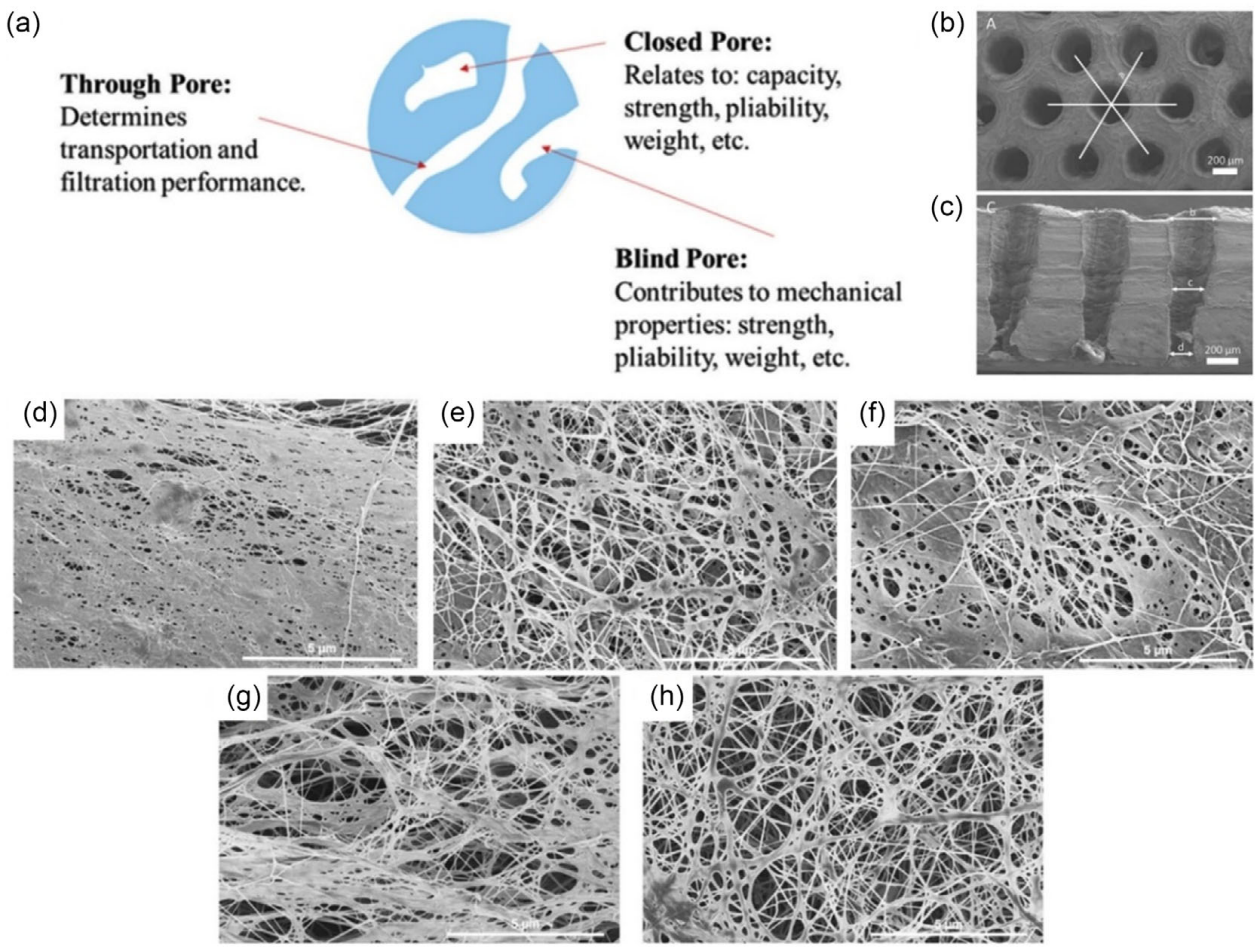
a) Illustration: Pore classifications and their effects on the overall mechanical and structural properties of BNC. Reproduced with permission.^[^ ^96^ ^]^ Copyright 2019, Royal Society of Chemistry. b) Top view (spherical pores) and c) cross‐sectional (trapezoid‐like) SEM images of a unidirectional laser‐perforated BNC scaffold with an overall honeycomb‐like macropore arrangement. b,c) Reproduced with permission.^[^ ^93^ ^]^ Copyright 2016, Springer Nature. d–h) SEM images of BNC membranes produced by cultures with: d) glucose, e) mannitol, f) sucrose, g) fructose, and h) glycerol carbon sources. Scale bar: 5 μm. d–h) Reproduced with permission.^[^ ^96^ ^]^ Copyright 2019, Royal Society of Chemistry.

A potential limitation in using unmodified BNC for TE scaffolds is that its small and heterogenous pore sizes may restrict cell migration and nutrient‐waste exchange (**Figure** **6**a). Nonetheless, this can be overcome by setting placeholders in the BNC synthesis culture or by post‐synthesis laser perforation (Figure 6b,c) to increase pore sizes.^[^ ^93^, ^94^ ^]^ Laser‐perforated BNC scaffolds showed a significant increase in cell‐seeding, aggrecan/collagen 1 and collagen 2/collagen 1 mRNA ratios, aggrecan and collagen 2 protein levels, and push‐out forces over time (for cell‐loaded scaffolds) when tested in a cartilage punch model.^[^ ^95^ ^]^ Recent findings further showed that the BNC porosity and morphology can be tuned simply by varying the carbon sources used in its culture media. Comparisons between static cultures with glucose, mannitol, sucrose, fructose, and glycerol revealed that glycerol‐ and fructose‐fed cultures produced BNCs with the highest porosities and pore surface areas (Figure 6d–h).^[^ ^96^ ^]^ This technique is advantageous in eliminating the need for post‐synthesis removal of placeholder materials and reducing the risk of BNC membrane perforation from physically intrusive methods.

#### 2.3.2 Bone TE

In bone TE, porous and mechanically viable scaffolds are required to support cell adhesion and proliferation of osteoblasts. Hydroxyl groups—intrinsically abundant on nanocellulose—has shown to aid bone regeneration through apatite deposition in the ECM.^[^ ^97^ ^]^ In cultures with bone‐marrow‐derived stem cells (BMSCs), the presence of CNCs can enhance the expression of osteogenic‐associated factors indicative of differentiation into bone cells.^[^ ^98^ ^]^ When coupled with alginate and gelatin in a 3D‐printable hydrogel, the physically crosslinked CNC‐based composite displayed superior mechanical and swelling properties, as well as better cell viability and mineralization with human BMSCs.^[^ ^99^ ^]^

Additionally, hydroxyapatite (HAP)—the main component of native bone tissue—is a common scaffold biomaterial used alongside nanocellulose, which has been well‐established to promote bone regeneration while being non‐cytotoxic to osteoblasts.^[^ ^92^, ^100^ ^]^ In an example, CNCs were mechanically strengthened by HAP coatings to form a hard matrix, which was further subject to layer‐by‐layer assembly using chitosan and hyaluronic acid (HA) to improve osteoblast cell viability (see ref. [101] for related works using the layer‐by‐layer technique).^[^ ^102^ ^]^ Extensive crosslinking methods are also employed to tune scaffold properties toward bone TE requirements. For instance, wood‐based CNF hydrogels were cross‐linked with gelatin to simulate collagen nanostructures in bone ECM.^[^ ^103^ ^]^ Variations of the cross‐linking methods/reagents including dehydrothermal treatment (DHT), hexamethylenediamine, and genipin were investigated for tuning the hydrogels’ mechanical properties and degradation rates to successfully support BMSC attachment, proliferation, and differentiation. Electrospinning is another popular approach that allows porosity control and easy integration of nanocellulose with other materials for scaffold reinforcement.[^97^, ^100^] Morphologically different nanocelluloses can even have positive mechanical synergy, demonstrated in a recent study wherein the addition of CNCs into electrospun CNFs significantly increased the overall nanofibers’ mechanical strength.^[^ ^104^ ^]^ This study further uncovered that aligned nanofiber scaffolds can induce directional BMSC growth and mineralization in vitro and orderly collagen assembly in vivo, forming highly organized bone structures.^[^ ^104^ ^]^ For BNC‐based scaffolds, a novel method to increase their pore sizes is by the addition of shredded agar into the bacterial synthesis media.^[100a]^ BNC‐HAP scaffolds fabricated this way showed increased mineralization of human osteoblast‐like cells (SaOs‐2) and cell migration.

#### 2.3.3 Neural TE

**Figure 7:**
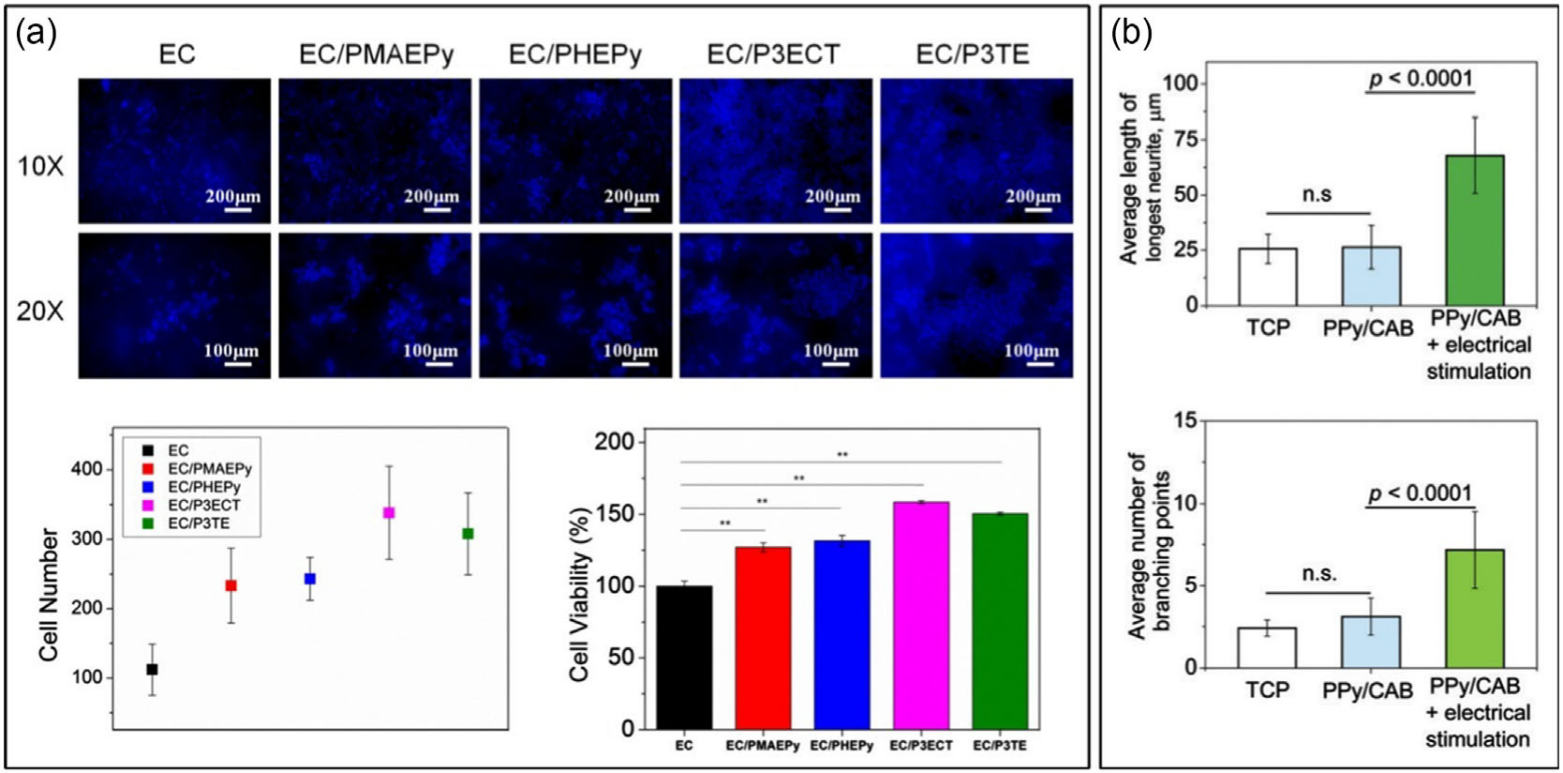
a) Staining with 4,6‐diamidino‐2‐phenylindole (DAPI) after 5 days of culture shows PC12 cell attachment and proliferation on electrospun nanocellulose (EC) modified with various conductive polymers. EC polymerized with poly(3‐(ethoxycarbonyl) thiophene) (EC/P3ECT) had the highest surface roughness and exhibited the greatest cell proliferation and viability. Reproduced with permission.^[^ ^110^ ^]^ Copyright 2021, Elsevier. b) Morphometric analysis of electrically stimulated and non‐stimulated human neuroblastoma cells (SH‐SY5Y) growing on randomly oriented polypyrrole‐coated cellulose acetate butyrate nanofibers (PPy/CAB). Electrically stimulated nanofibers show increased average neurite length (top graph) and number of branching points (bottom graph). TCP: control scaffolds, n.s.: not significant. Reproduced with permission.^[^ ^111^ ^]^ Copyright 2019, Royal Society of Chemistry.

In neural TE, conductive and oriented scaffolds are needed as conduits to guide neuron growth in an orderly fashion and direct cell fate for the treatment of neurological disorders/injuries. While scaffolds with high electrical conductivity are beneficial for nerve regeneration,^[^ ^105^ ^]^ many conductive polymers are mechanically fragile.^[^ ^106^ ^]^ To address this problem, nanocellulose can synergize with the conductive polymers to improve the overall mechanical properties of composite neural TE scaffolds while maintaining cell viability.^[^ ^107^ ^]^ Electrospun CNFs modified with conductive polymers via in situ polymerization showed improved scaffolding characteristics (e.g., porosity, thickness, conductivity) and favored PC12 cell development, as compared to unmodified electrospun CNFs.^[^ ^108^ ^]^ Surface topography is also important in providing contact guidance for the cellular reconstruction of neural tissue networks.^[^ ^109^ ^]^ Rougher surface morphologies were observed with an increase of conductive polymer nanoparticles incorporated into electrospun CNF scaffolds, which in turn enhanced PC12 cell adherence (**Figure** **7**a) and promoted protein adsorption—handy for adhering growth factors to assist neurogenesis.^[^ ^110^ ^]^ More interestingly, neural cell‐seeded conductive scaffolds can also be electrically stimulated to boost neuron growth with elongated and branched neurites (Figure 7b), as recently demonstrated by Elashnikov et al. using polypyrrole‐coated cellulose acetate butyrate nanofibers.^[^ ^111^ ^]^

3D bioprinting is robust in creating neural scaffolds with precise matrix structures and cell positioning—a necessity in replicating the complex neural ECM.^[^ ^112^ ^]^ Printed nerve scaffolds using alginate‐gelatin methacrylate (GelMA)‐BNC hydrogel with encapsulated RSC96 cells found the optimal 0.3% BNC concentration to impart outstanding mechanical and printing properties, cell adhesion, neurofactor expression, orderly cell growth along the scaffold axis, as well as satisfactory in vivo proliferation and vascularization.^[^ ^113^ ^]^ Moreover, Wei et al. have recently constructed nerve growth factor (NGF) encapsulated chitosan nanoparticles (CSNPs) in an oxidized BNC conduit.^[^ ^114^ ^]^ In vitro experiments demonstrated that such NGF@CSNPs/BNC nanocomposite promoted the adhesion and proliferation of Schwann cells, and further in vivo studies showed a successful repair of a sciatic nerve defect in rats within four weeks. It is worth additional note that the CSNPs also impart the nanocomposite with antibacterial activities, which will be particularly useful in actual clinical settings.

### 2.4 Antimicrobial Nanocelluloses

The growing challenges faced in infectious disease management along with existing issues of antibiotic resistance demands for alternative antimicrobial surface technologies to mitigate microbial (bacteria, fungi, viruses, algae, protozoa, etc.) infections. Though intrinsic antimicrobial activities of nanocellulose are lacking, it represents a prime natural carrier for antimicrobial agents due to its modification and functionalization flexibility, mechanical strength, and cytocompatibility. Capitalizing on its hydroxyl groups, surface functionalization via oxidation, esterification, and etherification are common methods to graft compounds that impart biocidal activity.^[^ ^115^ ^]^ Antimicrobial agents including biomolecules,^[^ ^116^ ^]^ antibiotics,^[^ ^117^ ^]^ and metal‐based nanoparticles,^[^ ^118^ ^]^ can also be added to nanocellulose. While demand for antibacterial nanocelluloses has been widely recognized and studied,[^24^, ^119^] their antifungal activity is relatively less evaluated, and fewer yet for antiviral examples. This section describes antimicrobial nanocellulose‐based materials evaluated with a wide range of microbes. Subsections follow the recent trends in strategies for the conferment of biocidal properties with appropriate antimicrobial agents.

#### 2.4.1 Physical Immobilization of Antimicrobial Agents

The high specific surface area, porosity, and versatile surface chemistry of nanocellulose make it conducive for the physical encapsulation and immobilization of antimicrobial agents.^[^ ^120^ ^]^ For example, the incorporation of polylactic acid (PLA)‐microencapsulated thymol, eugenol, and carvacrol into a BNC matrix for double barrier release achieved a fungicidal endpoint with half the dosage needed as compared to the free microcapsules.^[116c]^ The BNC matrix sustained fungicidal activity against *Candida albicans* by providing a longer diffusion path which maintained composite‐surrounding differential concentration for oil molecule release while minimizing oil denaturation. Thymol can also be impregnated in CNF structures in supercritical CO_2_ as a green approach.^[116d]^ The amount of adsorbed thymol increases with biomaterials’ specific surface area, which is largely attributed to hydrogen bonding between nanocellulose and the poorly soluble antimicrobial molecules.^[^ ^121^ ^]^ Other recent natural‐derived compounds physically incorporated in nanocellulose are plant derivatives from *Hemigraphis colorata* and *Chelidonium majus*,^[116e,116h]^ lactic acid bacteria postbiotics,^[116g]^ and arabinoxylans from brewers spent grain,^[116f]^ which successfully deactivated Gram‐positive (*Staphylococcus aureus, Listeria monocytogenes*) and Gram‐negative bacteria (*Escherichia coli, Pseudomonas aeruginosa*), as well as fungi (*C. albicans*).

In utilizing antimicrobial peptides, their poor aqueous solubility, low thermal stability, and susceptibility to enzymatic degradation under in vivo conditions can be circumvented when immobilized on nanocellulose. Catering to antimicrobial applications under specific environmental pH, nisin loading on TEMPO‐oxidized CNFs via electrostatic attraction was found to be highest at pH 3.8 and viable up to pH 8 under moderate ionic strength, making this composite suitable for bactericidal use in physiological conditions against *Bacillus subtilis* and *S. aureus*.^[116b]^ While the minimum inhibitory concentration (MIC) of biomolecule‐conjugated nanocellulose is sometimes higher than that of the free molecules due to decreased molecular spatial orientations after immobilization, the protection from molecule deactivation rendered by nanocellulose is an overall benefit in retaining longer biocidal activity.^[^ ^122^ ^]^ Combined entrapment of multiple antimicrobial biomolecules in nanocellulose matrices may also be needed to deliver a wide‐spectrum bactericidal activity for real‐life applications.^[^ ^122^, ^123^ ^]^

#### 2.4.2 Chemical Grafting and Surface Functionalization

A potential drawback of materials relying on the leaching of biocidal agents for antimicrobial action is their gradual decrease in effectiveness due to the loss of biocides. To maintain long‐term sterilization, chemical grafting or surface functionalization of biocides onto nanocellulose via covalent bonds is sometimes preferred. Chemical grafting of a conventional antibiotic, gentamicin, on CNF‐based lightweight sponges was achieved via enamine bond formation.^[^ ^117^ ^]^ Surface functionalized nanocellulose with aldehyde, carboxyl, and amine groups is also known to exhibit significant antibacterial activity.^[^ ^124^ ^]^

**Figure 8:**
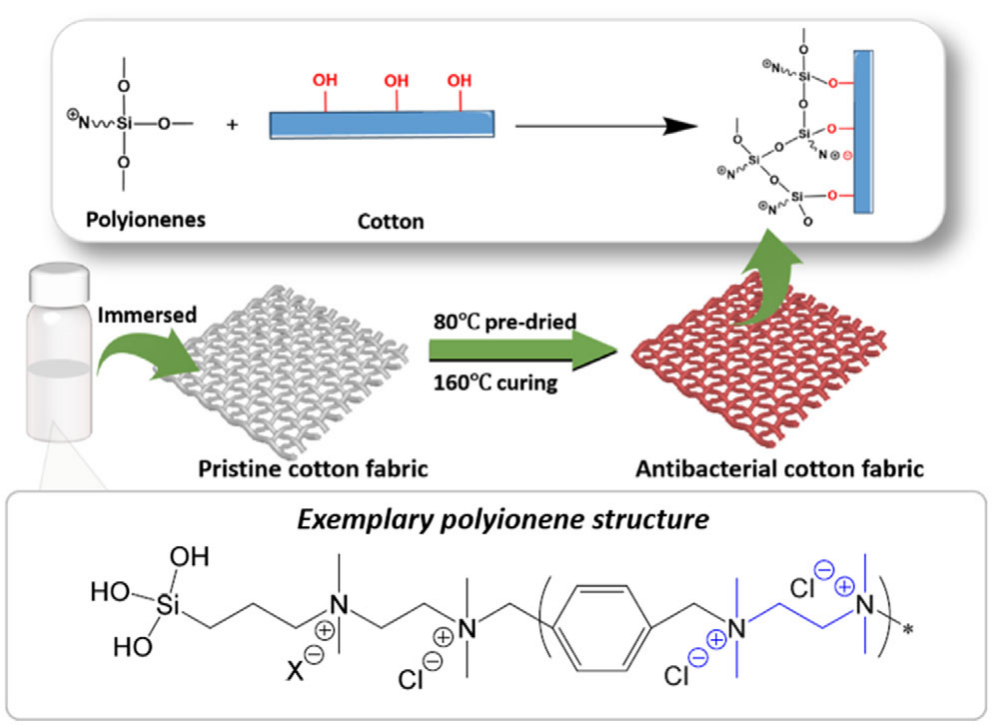
Schematic illustration of the coating process of silane‐functionalized polyionenes on cotton fabrics. The polymer grafting was achieved by the hydrolysis reaction between silane and hydroxyls abundantly available on cellulose. Reproduced with permission.^[^ ^126^ ^]^ Copyright 2022, Elsevier.

For aldehyde and carboxyl‐functionalized nanocellulose, bactericidal strength increases with the number of functional groups, which could be achieved through supplementary oxidation and even autoclaving.^[124d]^ Another common functionalization includes quaternary compounds such as pyridinium and imidazolium.^[^ ^115^ ^]^ For instance, bagasse‐derived CNFs were quaternized with 3‐chloro‐2‐hydroxypropyltrimethyl ammonium chloride in NaOH/urea solution and converted into triiodide form.^[^ ^125^ ^]^ The resulting quaternized CNF‐triiodide maintained peak antibacterial activity over 6 months against *E. coli* and *S. aureus*. Hexamethylenediamine (HMDA)‐functionalized CNFs as an antimicrobial additive to ring‐spun viscose yarn achieved up to 99% reduction of *S. aureus* and *Candida albicans*, and proved non‐irritant on rabbit skin.^[124c]^ In another recent example, Qiu et al. successfully coated cotton textiles with silane‐functionalized polyionenes (**Figure** **8**) which demonstrated potent bactericidal (>99.999%) and virucidal (7‐log PFU reduction) activities without causing any skin irritation.^[^ ^126^ ^]^ As the polyionene grafting was achieved by a hydrolysis reaction between silane and hydroxyls that are abundantly available on cellulose, this valuable coating strategy can be easily extended to other types of nanocellulose for antimicrobial applications.

#### 2.4.3 Metal‐Based Nanoparticles as Antimicrobial Agents

Metal‐based nanoparticles are another important family of antimicrobial agents with a long history of biocidal applications. The direct use of isolated nanometals in medical settings is, however, limited due to potential cytotoxicity to eukaryotic cells and environmental impact.^[^ ^127^ ^]^ However, their wide‐ranging biocidal properties can be harnessed when combined with biocompatible biopolymers such as cellulosic derivatives, which have shown to alleviate suspected toxicities and have been tested biocompatible.[^118^, ^119^, ^128^] They can be introduced into nanocellulose matrices as pre‐formed nanoparticles,^[118e]^ or via in situ generation approaches.^[118b,118c,118f]^

The latter is commonly employed in the fabrication of nanocellulose composites with silver nanoparticles (AgNPs)—the most widely studied metal antimicrobial.^[^ ^129^ ^]^ Nanocellulose‐AgNP composites are highly effective in killing various bacteria including multidrug‐resistant strains,^[118c]^ as well as fungi.^[118b,118f]^ In situ formation of AgNPs on nanocellulose usually involves immersing nanocellulose into a silver salt solution (usually AgNO_3_ or silver ammonium salts),^[118b−d,118f]^ before using appropriate reducing reagents to reduce the anchored Ag^+^ ions into AgNPs.^[^ ^130^ ^]^ Morphology and resulting antimicrobial activity of generated AgNPs are highly dependent on their synthesis conditions. Nanocellulose with smaller AgNPs—formed using stronger reducing agents or higher (to an extent) reaction pH[^118^, ^131^]—displayed stronger antibacterial activity due to an overall larger binding surface area. Recent foci in this area include green fabrication strategies for antimicrobial nanocellulose‐metal composites, in which green reducing agents (e.g., ascorbic acid,^[118c]^ glucose,^[118g]^ ethylene glycol^[118h]^) have been employed in nanocellulose‐AgNPs fabrication. Still, these methods generally involve harsh chemicals in the nanocellulose fabrication steps,^[118g]^ or energy‐intensive heating in AgNPs generation.^[118b]^

Metal–oxide nanoparticles can be similarly formed in situ with nanocellulose via ion reduction. A recent study optimized fabrication methods for BNC aerogels decorated with copper oxide (CuO) and zinc oxide (ZnO) nanoparticles, synthesized from CuSO_4_⋅5H_2_O and Zn(NO_3_)_2_⋅6H_2_O, respectively.^[118i]^ Antibacterial activity against *Klebsiella pneumonia* and *S. aureus* was compared between BNC aerogels fabricated with Ag, CuO, and ZnO nanoparticles, which confirmed its correlation to the amount and size of metal nanoparticles present. In alternative methods, ZnO nanoparticles can be first prepared via precipitation and subsequently coated onto CNFs.^[118j]^ Similarly, the antimicrobial strength of ZnO pre‐formed nanoparticles varies with their size and shapes, which can be tuned by fabrication approaches used and parameter control (e.g., temperature, pH, etc.).^[^ ^132^ ^]^ Other antimicrobial metal derivatives include Ca^2+^ and Cu^2+^ ion‐crosslinked CNF hydrogels which decelerated *Staphylococcus epidermidis* colonization and impeded biofilm formation of *P. aeruginosa*.^[^ ^133^ ^]^

**Figure 9:**
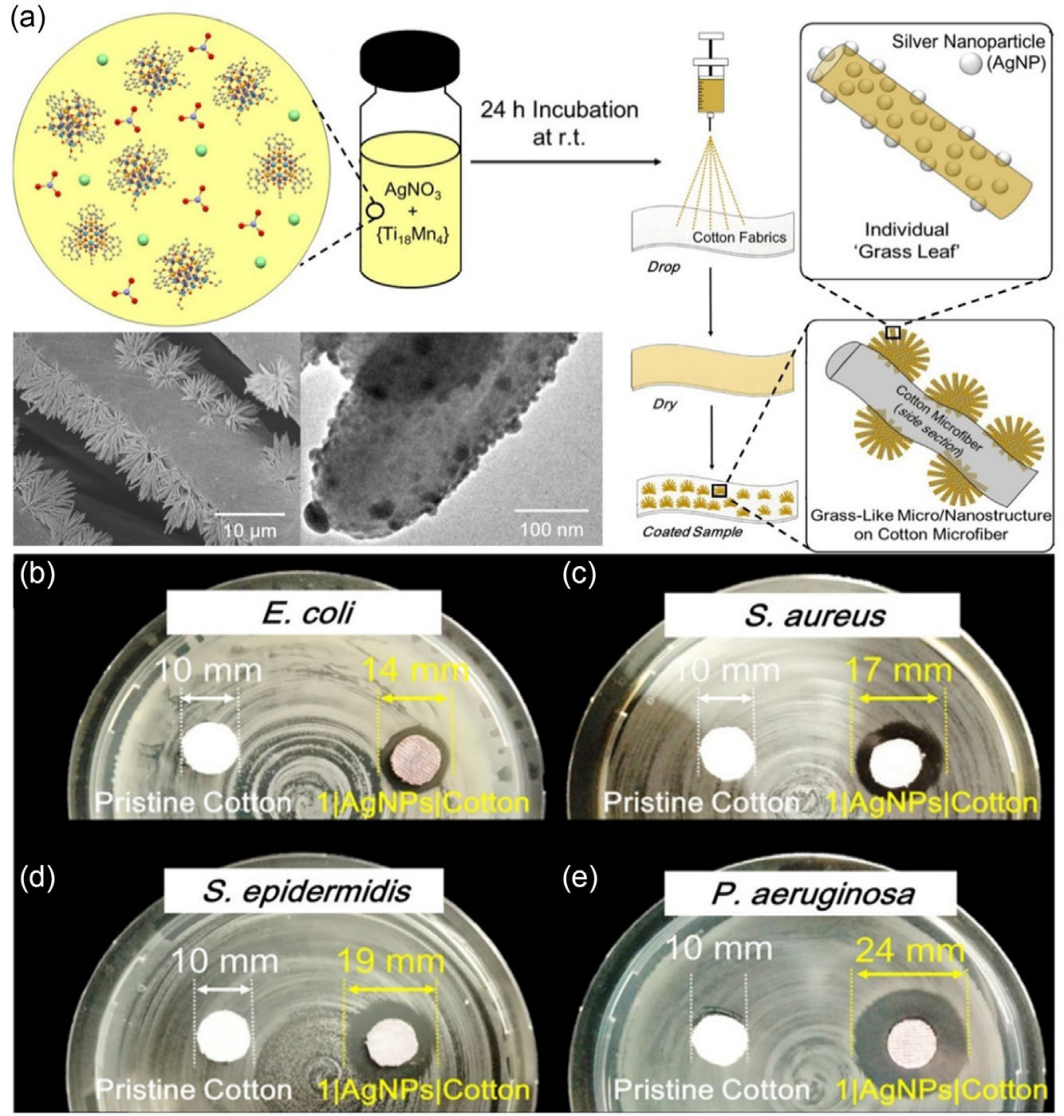
a) Schematic illustration of the preparation steps for in situ self‐assembly of heterogenous grass‐like coatings on cotton microfibers. Field‐emission scanning electron microscopy (FESEM) images show AgNPs’ distribution on the surfaces and embedment within individual “grass leaves”. b–e) Bacterial inhibition activity of unmodified cotton and TOC‐AgNP embedded cotton microfibers on agar plates of: b) *Escherichia coli*, c) *Staphylococcus aureus*, d) *Staphylococcus epidermidis*, and e) *Pseudomonas aeruginosa*. a–e) Reproduced with permission.^[^ ^137^ ^]^ Copyright 2020, American Chemical Society.

Besides metal cations and nanoparticles, single molecular metal‐oxo clusters (MOCs) of the type [M_*x*_O_*y*_(OR)_*z*_] (OR = alkoxide) emerged in recent years as a unique molecular mimic of metal oxides for antimicrobial coating applications.^[^ ^134^ ^]^ Such MOCs are structurally versatile, synthetically controllable and have displayed useful antibacterial and UV‐blocking properties,^[^ ^135^ ^]^ making them viable structural models for metal‐based nanoparticles.^[^ ^136^ ^]^ These MOCs can further synergize with other antimicrobial components to form intricate nanostructures with enhanced biocidal activity. In a recent report, a one‐step “drop‐and‐dry” approach using a precursor solution of [Ti_18_MnO_30_(OEt)_20_(MnPhen)_3_] (Phen = 1,10‐phenanthroline) and AgNO_3_ facilitated the in situ self‐assembly of heterogeneous grass‐like nanostructures on cotton cellulose microfibers (**Figure** **9**a).^[^ ^137^ ^]^ AgNPs were found to be distributed on the surface and interior of the nanostructures, and the resultant microfibers showed prominent antibacterial activity and UV‐blocking capabilities. Such a coating method should be easily transferrable to nanocellulose substrates, especially the highly porous CNFs and BNC, for antimicrobial coating purposes, yet the biocompatibility and cytotoxicity of such MOCs have to be addressed in future studies for practical application.

A potential issue in this area is the demonstrated ability of bacteria to develop resistance to AgNPs by producing flagellin, an adhesive protein of the bacterial flagellum, which causes colloidal AgNPs to aggregate, hindering their biocidal effectiveness.^[^ ^138^ ^]^ Similar resistance mechanisms could also occur for other metal nanoparticles. While it cannot be subverted by steric stabilization of AgNPs with surfactants or spacer polymers to prevent aggregation, the developed bacterial resistance can be suppressed by inhibiting flagellin production using pomegranate rind extract.^[^ ^138^ ^]^ Alternatively, ionic metals can instead be employed as the main antibacterial agent against nanoparticle‐resistant bacterial strains; ionic silver was unaffected by the colloidal particle aggregation‐based bacterial resistance mechanism. However, reports of bacteria resistance to ionic silver have suggested mechanisms involving the reduction of Ag^+^ to the less toxic states or an active efflux of Ag^+^ from the cell by P‐type adenosine triphosphatases and chemiosmotic Ag^+^/H^+^ antiporters;^[^ ^139^ ^]^ further investigations are required for the validation of microbial resistance mechanisms and to develop counteracting solutions, such as combined use of multiple metal‐based antimicrobial agents.

#### 2.4.4 Viral Deactivation and Removal

Nanocellulose structures for the loading of antiviral drugs are commonly fabricated with nanoparticulate forms for efficient drug delivery and subsequent release. The antiviral drug acyclovir was complexed in nanoparticles of carboxymethyl cellulose acetate butyrate via two tested methods—conventional precipitation, and rapid precipitation in a multi‐inlet vortex mixer.^[^ ^140^ ^]^ Recent trends in virus removal converge toward filtration methods utilizing the size‐exclusion principle, which presents the advantages of being non‐biologically interfering and non‐immunogenic. Parameters that affect virus filtration efficiency include filter porosity and pore sizes, thickness, surface chemistry, and pH of the filter media. Concept studies established the viability of pristine CNFs as filter membranes for the swine influenza virus (SIV, average particle size of 80–120 nm) as a model strain.^[^ ^141^ ^]^ It achieved a log_10_ reduction value (LRV) of ≥6.3, matching the operational values of industrial synthetic polymer virus removal filters. Nanocellulose filters were subsequently tested for viral filtration of various biological feed media such as in the bioprocessing of human intravenous immunoglobulin (IVIG),^[^ ^142^ ^]^ Dulbecco's modified Eagle's medium (DMEM), and Luria‐Bertani medium (LBM).^[^ ^143^ ^]^ These respective studies achieved filtration at high volumetric loads for viruses including ΦX174 (≈32 nm) and MS2 (23–28 nm) bacteriophages. Notably, curcumin‐loaded CNCs were proposed as a possible inhalable nano‐therapeutic for the treatment of the prevailing coronavirus disease 2019 (COVID‐19) due to its potential inhibitory effects on the positively charged glycoproteins of the severe acute respiratory syndrome coronavirus 2 (SARS‐CoV‐2) virus.^[^ ^144^ ^]^

### 2.5 Wound Dressing and Healing

Wound dressings used in promoting tissue healing ought to be sufficiently hydrophilic to facilitate gas/fluid exchange, absorption of excess exudates, and manage fluid loss. Drug delivery, TE, and antimicrobial techniques (described earlier) are often integrated with dressing designs to maximize healing effectiveness and efficiency.

**Figure 10:**
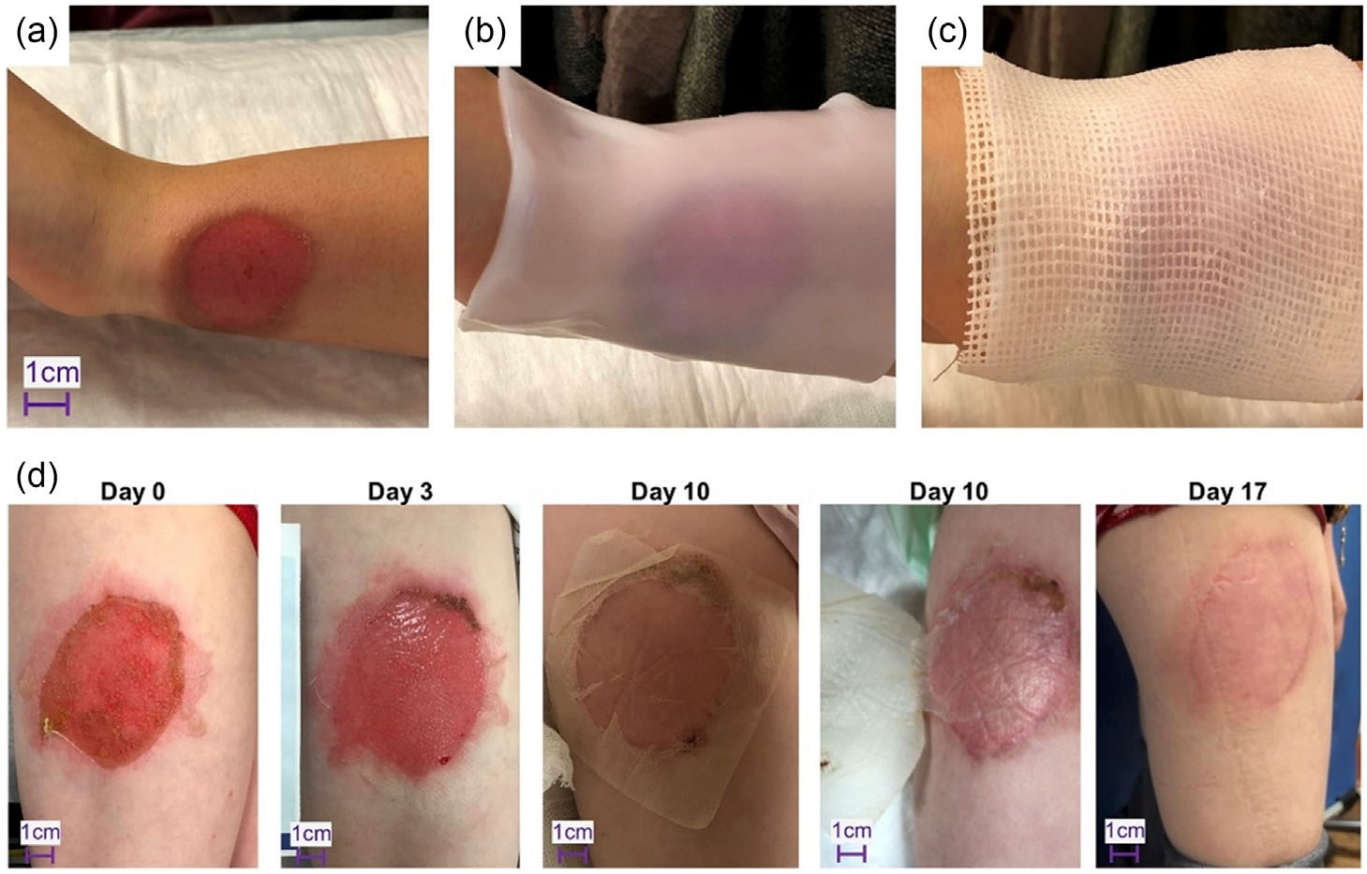
a–c) BNC dressing application procedures: a) Initial wound assessment and disinfection, b) BNC dressing application, c) overlaid fatty gauze to maintain moisture. d) Wound healing progress of a 2% burn on the outer thigh of a 3‐year‐old child. Day 0: initial presentation, days 3 and 10: dressing changes and wound assessments, day 17: final follow‐up visit. BNC became transparent and was easily removable on day 10 due to full re‐epithelialization. a–d) Reproduced with permission.^[^ ^147^ ^]^ Copyright 2021, The Authors, published by Medicalhelplines.com Inc (3M) and John Wiley & Sons Ltd.

In view of its green biosynthesis and ideal physicomechanical attributes, pristine BNC has been widely used for wound healing in the past decade.^[^ ^145^ ^]^ In the treatment of burn wounds, the high water content of BNC is extremely conducive for cooling of burns via evaporation. Ex vivo models of burnt skin explants, when treated with BNC dressings, showed a significantly decreased intradermal temperature, as well as higher cell vitality, less necrosis, and less dermal–epidermal separation observed on histological assessments.^[^ ^146^ ^]^ Because BNC cools burn wounds via evaporation, the need for pre‐cooled dressings is decreased and its corresponding drawbacks of reduced tissue perfusion and re‐epithelialization caused by sudden temperature drops are avoided. Clinical trials on children with partial thickness burns revealed full re‐epithelialization within 7–17 days and were >95% healed for most patients on day 10 of BNC application (**Figure** **10**).^[^ ^147^ ^]^ In addition, BNC maintained an optimal moist environment which required less frequent dressing changes. Together with its ease of removal without inducing secondary or collateral tissue damages—an important requirement in wound dressings^[^ ^148^ ^]^—and facile cuff design (Figure 10a–c), pain levels experienced could be significantly reduced. In another clinical trial, the use of BNC for healing tympanic membrane perforation wounds saw a large reduction in operational time and costs compared to conventional methods using autologous temporal fascia, while being just as effective in wound closure as conventional treatments.^[^ ^149^ ^]^

Incorporation of a secondary hydrophilic material can improve the performance of BNC dressings, including but not limited to better water retention and reduced dressing changes.^[^ ^150^ ^]^ Alginate is an example; BNC/alginate wound dressings by Sulaeva et al. not only supported the impregnation of antimicrobial agents for preventing wound infections, its fabrication was also tested in industrial facilities and showed potential for large‐scale production.^[^ ^150^ ^]^

**Figure 11:**
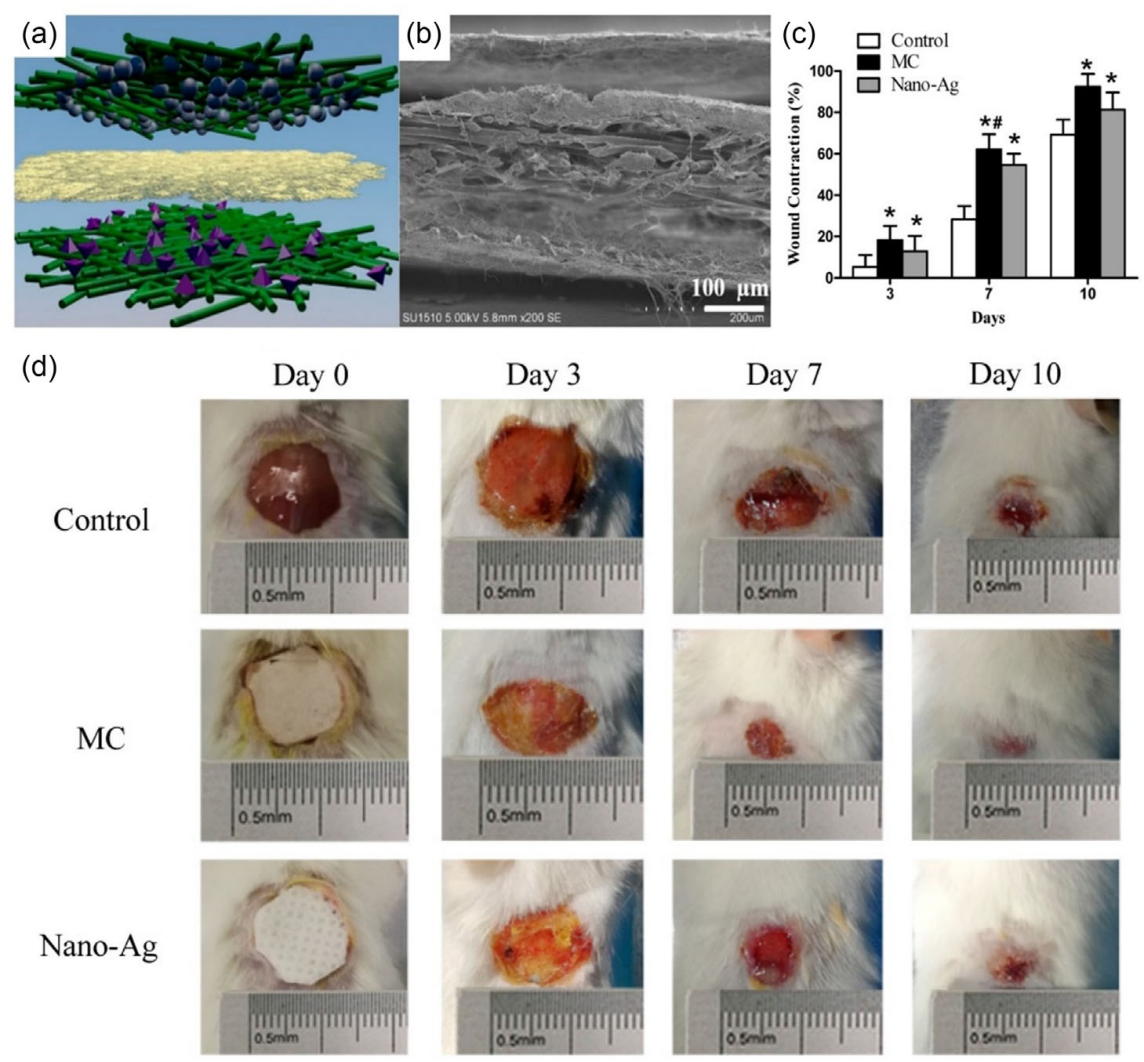
a) Illustration of antibacterial layer (top), reinforcement BNC layer (middle), and vaccarin healing promotion layer (bottom) in a multilayered composite membrane. b) Cross‐section SEM images of fabricated multilayer composite. c) Wound contraction rate of mice skin wounds treated with sterile gauze (Control), multilayered composite (MC), and Nano‐Ag. Evaluated on days 3, 7, and 10. * represents a statistically significant difference from the Control group at *p* < 0.05, # represents a statistically significant difference from the Nano‐Ag group at *p* < 0.05. d) Photographic images of respectively treated wounds on days 0, 3, 7, and 10. a–d) Reproduced under the terms of the CC‐By Creative Commons Attribution 4.0 International license (https://creativecommons.org/licenses/by/4.0).^[^ ^155^ ^]^ Copyright 2020, The Authors, published by MDPI.

An ideal wound dressing should prevent microbial infiltration into the wound area which may cause infections and hamper the healing process. Incorporation of antimicrobials (see Section 2.4) including bioactive molecules and metals are effective in further improving pristine nanocelluloses’ wound healing capacities.^[^ ^151^ ^]^ BNC/ZnO composites evaluated on mice burn wounds showed broad‐spectrum antibacterial activity against *E. coli, P. aeruginosa*, *S. aureus,* and *Citrobacter freundii* as well as significantly faster healing than treatments with pristine BNC.^[^ ^152^ ^]^ Factors affecting the loading and release efficiency of antiseptics in BNC (e.g., solution molecular weight) were analyzed and discussed by de Mattos et al.^[^ ^153^ ^]^ BNC as an additive to chitosan/gelatin dressings not only enhanced its mechanical properties, but also demonstrated the highest wound contraction on day 15 of in vivo rat wound treatment when compared with a negative control and the chitosan/gelatin film alone.^[^ ^154^ ^]^ Further incorporation of tannic acid could increase fibroblast proliferation and epidermis/dermis regeneration. Notably, a multilayered composite (MC) membrane (**Figure** **11**a,b) comprising: 1) an antibacterial layer of electrospun zein/ethyl cellulose nanofibers loaded with antibacterial photosensitizer protoporphyrin, 2) a reinforcement BNC layer, and 3) a healing promotion layer of vaccarin was fabricated and tested on mice skin wounds in comparison with sterile gauze controls and commercial Nano‐Ag dressings (Figure 11d).^[^ ^155^ ^]^ The MC dressing saw a 92.4% wound contraction on day 10 of treatment—higher than the commercial Nano‐Ag (Figure 11c), while also outperforming in wound exudate reduction and avoiding induced trauma during dressing removal. Similar combinations of multifunctional biomaterials have seen increasing progress toward synergistic and effective wound healing.^[^ ^156^ ^]^

Although BNC is popularly used here as it facilitates fluid exchange and has good moisture retention, plant‐derived nanocellulose also demonstrated similar effectiveness when tested against commercial BNC membrane Membracel.^[^ ^157^ ^]^ Fibrous CNFs have characteristics alike BNC which can provide a suitable porous environment for wound healing, while the nanoparticulate CNCs are usually assembled into hydrogels, or blended with other materials and processed into films or ointments.^[^ ^158^ ^]^ In polyvinyl alcohol (PVA)/CNC hydrogels prepared by freeze‐thawing, the addition of CNCs controlled the hydrogel's porous morphology, maintained its transparency, enhanced its thermal and mechanical stability, and could tune its water vapor transmission rate to lie within the favorable range for wound dressings.^[^ ^159^ ^]^ They functioned well as protective barriers against the various bacterial pathogens present in wounds. Diosgenin‐conjugated carboxyl CNC hydrogels crosslinked with genipin were loaded with the antibiotic neomycin to form a bacteria‐inhibiting and impenetrable wound dressing.^[^ ^160^ ^]^ In a pioneer clinical study of CNF dressings for skin graft donor site treatment, wood‐derived CNF dressings are attached and adhered securely on donor wounds till site renewal.^[^ ^161^ ^]^ On top of outperforming the commercial dressing Suprathel in its healing rate, they also self‐detached from epithelialized skin—a desirable feature in ideal wound dressings. This dressing has since been commercialized (FibDex) and its effectiveness was ascertained in further clinical trials.^[^ ^162^ ^]^ Recent additive manufacturing advances in this area include the formulation of TEMPO‐oxidized CNFs with GelMA into a printable hydrogel,^[^ ^163^ ^]^ which offers tailoring of dressing morphology towards the ideal for treating different wound types.

An advanced wound management strategy is also to include active drug components in dressings and capitalize on its controlled delivery to accelerate wound healing. A polydopamine/tetracycline hydrochloride (TH)‐loaded CNF hydrogel displayed capabilities of on‐demand drug release of TH molecules upon near‐infrared (NIR) irradiation or at low pH environments for tailored wound healing.^[^ ^164^ ^]^ For the management of wound inflammation, BNC was loaded with lipophilic nanoemulsions of anti‐inflammatory *Boswellia serrata* extract via water exchange.^[^ ^165^ ^]^ The loaded BNC could be gently sterilized by electron beam irradiation and its formulations were varied to provide the desired skin penetration levels of the plant extract for treating superficial or chronic skin diseases. Therapeutic molecules can also be loaded in nanocellulose carriers which protect and maintain their functions, and subsequently release them controllably. Investigations on a Ca^2+^‐crosslinked wood‐based CNF hydrogel as a therapeutic protein carrier revealed electrostatic interactions between proteins and the negatively charged hydrogel as the main mechanism for protein loading and release, whereby proteins of larger sizes and positive charge sustained a slower release.^[^ ^166^ ^]^ Further in vivo tests on dermo‐epidermic full‐thickness rat wound models showed remarkably accelerated healing and epithelialization with the CNF hydrogel treatment when compared to a cotton gauze control, whereby a fully healed epithelium was observed at day 25 of the CNF treatment.^[^ ^167^ ^]^ Additionally, an advanced dual‐release system for synergistic drug delivery and wound healing was exhibited by core‐shell microparticles of CNC/vascular endothelial growth factor (VEGF) cores and alginate/doxycycline hydrochloride (DH) shells.^[^ ^168^ ^]^ The antibacterial shell effectively reduced early‐stage inflammation in wound healing through its quick‐release of antibiotics and thereafter promoted angiogenesis through VEGF release from the CNC core.

### 2.6 Medical Implants

A general difference between implants and TE scaffolds (discussed in Section 2.3) is in their durability and bioresorbability requirements. Medical implants, as described in this section, replace lost or damaged body tissues (often permanently), and are expected to maintain their intended function for the long‐term whereas TE scaffolds provide temporary support to guide cell repopulation and are usually bioresorbable.

**Figure 12:**
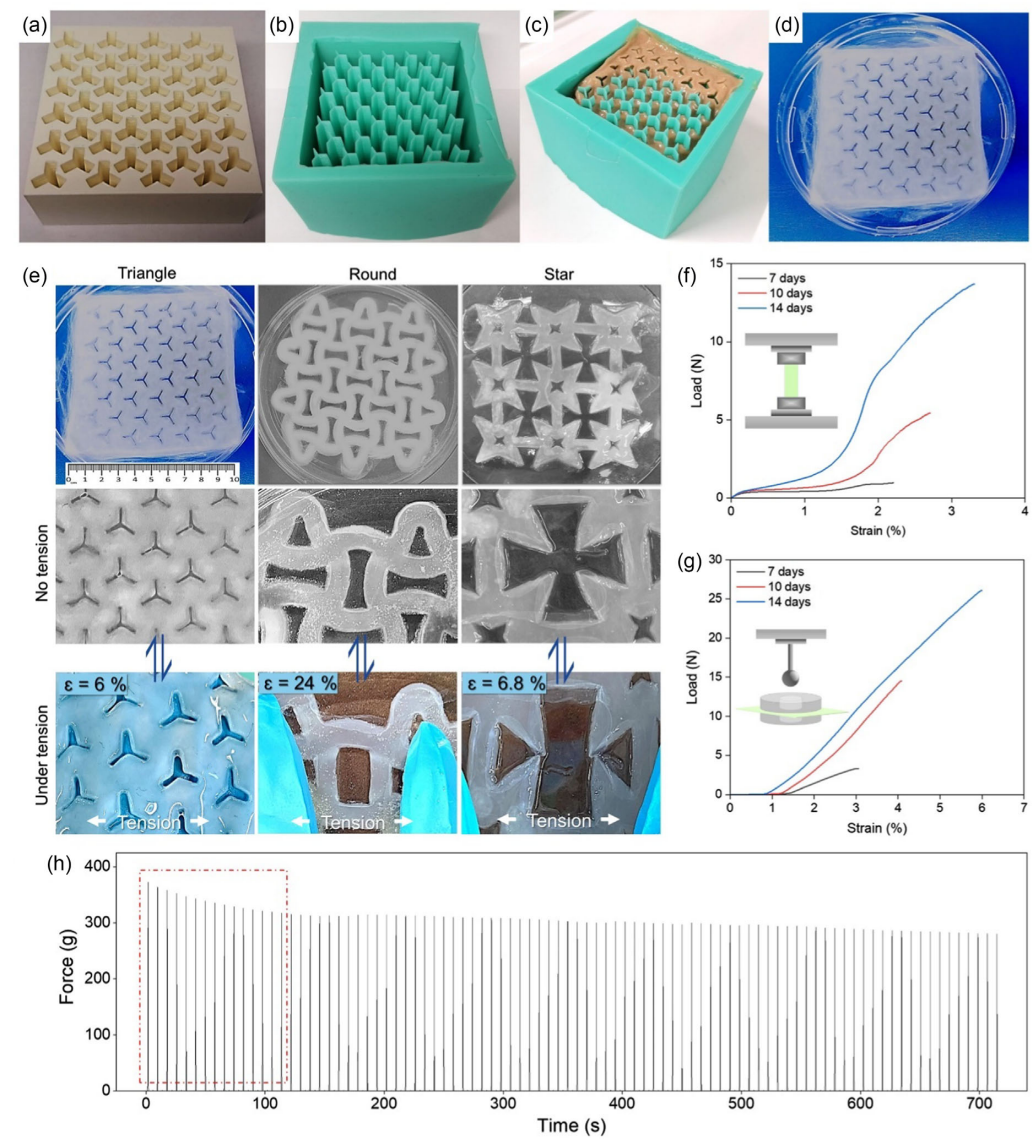
a) 3D‐printed auxetic polylactic acid (PLA, positive) models. b) Cast silicon (negative) molds yielded after full curing for about 6 h in room condition. c) BNC structure formed on culture medium surface on day 10 of culturing in silicon molds. d) BNC film removed and purified to obtain the final auxetic BNC mesh (10 cm × 10 cm). e) Triangle, round, and star‐patterned auxetic BNC meshes obtained after 10 culture days. Meshes exhibit auxetic properties and reversible structural expansion under tension. Poisson's ratios: *ν* = −0.19 (triangle), *ν* = −0.36 (round), and *ν* = −0.13 (star). f) Load–elongation profiles of BNC meshes cultured for 7, 10, and 14 days when subjected to tensile tests and g) Ball burst strength tests. h) Cyclic burst strength at 3% strain using 10 day cultured BNC mesh with sustained performance after 100 loading/unloading cycles. a–h) Reproduced under the terms of the CC‐BY Creative Commons Attribution 4.0 International license (https://creativecommons.org/licenses/by/4.0).^[^ ^173^ ^]^ Copyright 2022, Elsevier.

The excellent mechanical properties of nanocellulose‐based materials are optimal for the replacement of faulty tissues which undertake significant mechanical loadings.^[^ ^169^ ^]^ Stress‐relaxation studies on cell‐supportive CNF nanocomposites at simulative human body conditions (37 °C, 95% relative humidity) showed stable performance under cyclic loading/unloading and mechanical resemblance to actual ligaments and tendons.^[^ ^170^ ^]^ Similarly, CNFs as hydrogel reinforcements contributed to material recovery after compressions and straining, and were proposed as replacements for the nucleus pulposus of intervertebral discs.^[^ ^171^ ^]^ An important requirement of supportive mesh implants is to maintain their porosity and mesh openings under stress so that tissue ingrowth is not disrupted.^[^ ^172^ ^]^ Auxetic BNC structures present the advantage of structural recovery after dynamic deformations due to body movements, as well as being mechanically and geometrically tunable via BNC culture conditions.^[^ ^173^ ^]^ Meshes with prescribed auxetic geometries and infill density were fabricated by bacteria culturing in silicon molds derived from 3D‐printed guides (**Figure** **12**a–d).^[^ ^173^ ^]^ After 10 days of culturing, they exhibited reversible expansion under tension, with different (negative) Poisson's ratios for each auxetic geometry (Figure 12e). Mechanical strength of BNC meshes increased from 48 to 456 MPa with culture duration (Figure 12f) and over 87% stability after 100 burst loading/unloading cycles (Figure 12h), showing comparability with human muscular tissue and potential long‐term viability for implantation in sites which experience routine dynamic loading.

A universally versatile method for coating BNC on 3D surfaces proposed the deposition of polydopamine on objects to promote bacteria attachment for subsequent in situ synthesized BNC networks.^[^ ^174^ ^]^ As opposed to standard templating methods of culturing BNC in 3D shapes, direct growth and immobilization of BNC on surfaces can achieve more complex geometries and cellular architectures. The enhanced water content, conformability, and perpendicularly grown BNC fiber orientation confer a highly lubricious surface with high mechanical stability and energy dissipation capabilities that present excellent potential as biocompatible coatings for load‐bearing medical implants.^[^ ^174^ ^]^

**Figure 13:**
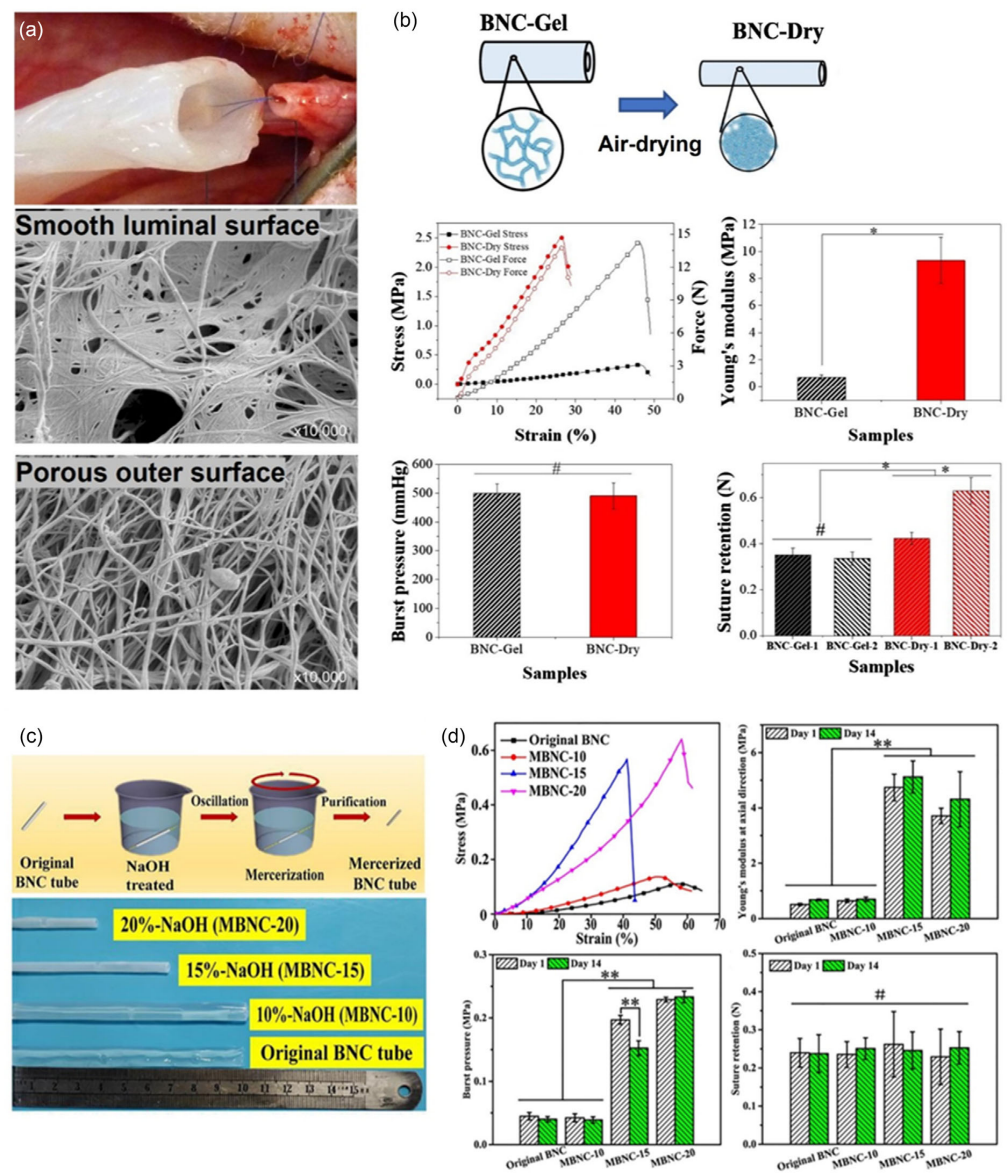
a) Surgical implantation of BNC small‐diameter vascular tubes. SEM images of inner (smooth) and outer (porous) surfaces. Reproduced with permission.^[^ ^175^ ^]^ Copyright 2018, Elsevier. b) Comparisons of stress, elastic modulus, burst pressure, and suture retention between wet BNC hydrogel small‐diameter tubes (BNC‐Gel) and air‐dried BNC tubes (BNC‐Dry). 5‐0 Dacron suture was used for BNC‐Gel‐1 and BNC‐Dry‐1. 10‐0 nylon suture was used for BNC‐Gel‐2 and BNC‐Dry‐2. #: *p* < 0.05, *: *p* < 0.01. Reproduced with permission.^[^ ^180^ ^]^ Copyright 2021, Elsevier. c) Preparation scheme of alkaline mercerization treatment. BNC tubes of varying dimensions are obtained when mercerized at different NaOH concentrations. d) Comparisons of stress, elastic modulus, burst pressure, and suture retention between tubes mercerized at different NaOH concentrations. *: *p* < 0.05, **: *p* < 0.01, #: *p* > 0.05. c,d) Reproduced with permission.^[^ ^181^ ^]^ Copyright 2022, Elsevier.

Synthetic vascular grafts present an alternative to autologous vascular transplantation which bypasses the latter's limitations in availability and surgical derivation. Despite the clinical success of large‐diameter vascular prosthetics (>8 mm inner diameter), artificial small‐diameter grafts (<6 mm) face limitations of poor patency due to risks of thrombosis.^[^ ^175^ ^]^ A multitude of nanocellulose‐based synthetic blood vessels has been proposed following observations of good haemocompatibility and mechanical suitability.^[^ ^176^ ^]^ Recent efforts focused on improving the long‐term patency of small‐diameter grafts, in which BNC is extensively studied in.^[^ ^177^ ^]^ It was established that a smooth luminal surface was important for the bio‐ and haemocompatibility of BNC vascular grafts, as well as to reduce blood coagulation and adhesion of thrombocytes and leukocytes.^[^ ^178^ ^]^ Synthetic BNC tubes with diminished wall thickness, smooth inner surface, and porous outer zones were proposed and verified (**Figure** **13**a) to improve graft patency by reducing thrombogenic potential and facilitating easier cell immigration along the outer layer.^[^ ^175^ ^]^ However, dual antiplatelet treatment was necessary to maintain long‐term patency; grafts were occluded at 9 months post‐implantation otherwise. Evaluations on BNC tubes fabricated by three different bioreactors also corroborate the need for smooth luminal surfaces and good mechanical properties (tensile strength, burst pressure, suture retention).^[^ ^179^ ^]^ Still, they were not mechanically comparable to a clinically used polytetrafluoroethylene prosthesis. To solve this, air‐drying shows potential for strengthening BNC conduits and improving suture retention (Figure 13b).^[^ ^180^ ^]^ Notably, mercerization (alkaline treatment of BNC, Figure 13c) is a recent development toward fine‐tuned reduction of BNC tube thickness without sacrificing its mechanical properties—they were instead enhanced (Figure 13d).^[^ ^181^ ^]^ Composition of BNC with heparin, silk fibroin, and chitosan could also improve the anticoagulant properties and stimulated endothelialization^[^ ^182^ ^]^ while toxicology studies on chitosan‐modified BNC established a lack of adverse reactions when implanted in animal models^[^ ^183^ ^]^; future studies could examine the long‐term patency rates of such composite vascular grafts. While the aforementioned tubular BNC prostheses are mostly fabricated by bacteria in silicone‐tubed bioreactors,^[^ ^179^, ^184^ ^]^ other interesting methods proposed are electrospinning,^[^ ^185^ ^]^ layer‐by‐layer fabrication,^[^ ^175^ ^]^ and rolling of BNC layers with microfluidic cell patterning.^[^ ^186^ ^]^

Heart valve replacement is the currently practiced procedure for the end‐stage treatment of several aortic and mitral valve diseases. The current state of the art in clinical use are mechanical valves and bioprosthetic valves, which endure continual transient high‐pressures and bending stress cycles upon surgical implantation. Mechanical heart valves are durable; however, its use is limited by the risks of induced thromboembolism, hemolysis, and hemorrhage.^[^ ^187^ ^]^ They also require chronic anticoagulation maintenance, and for pediatric patients, multiple valve resizing surgeries.^[^ ^188^ ^]^ In contrast, bioprosthetic valves usually do not require chronic anticoagulation treatments, but they are relatively short‐lasting due to mineralization and tearing of the tissue material.^[^ ^187^ ^]^ While clinical studies have not determined the better of the two due to demographical and statistical confounders,^[^ ^188^, ^189^ ^]^ ideal materials for heart valve construction should be mechanically durable yet hemocompatible, toward which natural polymeric materials could provide viable solutions. Though studies on nanocellulose‐based artificial heart valves are still lacking, the use of nanocellulose to modify other materials has been demonstrated in the literature.^[^ ^190^ ^]^ Together with the expected rise in computational simulations and machine learning for in silico modeling of nanocellulosic materials, guidelines for constructing heart valves with combined mechanical and biological advantages can be foreseen in the near future.

## 3 Summary and Future Perspectives

From both academic and economic perspectives, nanocellulose, a gift from nature, represents a prominent family of sustainable materials for cutting‐edge biomedical technologies. The present review examines the importance and recent advancements of nanocellulose materials toward biomedical uses in themes including biomolecule immobilization, programmable drug delivery, TE, antimicrobials, wound healing, and medical implants. The significance of nanocellulose in these systems is discussed in their fabrication methodologies and applicability. Notable works are highlighted; challenges faced are reviewed with possible suggestions. In materials for biomedical applications, a recurring subject of study is the validation of material biocompatibility and non‐cytotoxicity. While a vast majority of nanocellulose works present negligible toxicity, it is worth noting that plant‐derived compounds contain some level of endotoxins—a measure regulated by the Food and Drug Administration, U.S. (FDA) in biomedical and food‐grade applications. CNC and CNF lengths were also found to have implications on cytotoxicity and inflammatory response in some cases.^[^ ^191^ ^]^ Hence, nanotoxicity monitoring is particularly essential for biomedical safety. The growing advances in artificial intelligence and machine learning algorithms for modeling nanomaterial and nanocomposite toxicity enable insights into biological interactions of nanomaterials where it is difficult to investigate via bio‐experimental methods.^[^ ^192^ ^]^ Examples include machine learning models for predicting protein corona compositions of nanocellulose and metal nanoparticles to determine their behaviors in biological systems,^[^ ^193^ ^]^ as well as using computational models to compensate for experimental limitations such as the biological variability of animal test subjects.^[^ ^194^ ^]^ Overall, in tandem with methods for producing ultra‐pure nanocellulose, the integration of artificial intelligence paves a direction toward ensuring adequate biosafety in cellulose nanomaterials and nanomedicines.

By virtue of its bioavailability, green biosynthesis (for BNC), and favorable physicochemical characteristics, the production of nanocellulose and derivatives has attracted a sizeable proportion of research works worldwide. Currently, large‐scale production of plant‐based nanocellulose (i.e., CNFs and CNCs) has been tested industrially at several demonstration plants in Europe, Japan, Canada, and the US. However, manufacturing processes are generally energy‐demanding, and their potential environmental impact remains unclear.^[^ ^195^ ^]^ In comparison, despite considerable recent progress, production of BNC still remains limited, mainly hindered by the costly support needed for bacteria culture and its relatively low production yield. Production upscaling may also further impair its yield as BNC‐producing bacteria are highly sensitive to changes in their culture conditions.^[^ ^196^ ^]^ Life cycle assessments (analyses of nanocellulose extraction, synthesis into useful materials, transportation, consumption, and disposal)^[^ ^197^ ^]^ should also be conducted to monitor the environmental impacts of nanocellulose use. Nonetheless, the exponentially growing knowledge base and extensive guidelines proposed for green and efficient nanocellulose production offer an optimistic trend for its future in industrial‐scale applications.^[^ ^198^ ^]^ This will be surely further accelerated by the advancements in artificial intelligence for nanomaterial modeling to present valuable guidance for the automation and optimization of nanocellulose production by reducing experimental costs and time. To this end, various advanced simulation and analytical machine learning models were proposed for uncovering structure–property relationships and predicting the mechanical behavior of novel nanocellulose composites,^[^ ^199^ ^]^ providing guidelines for fabricating nanocellulose materials with desired characteristics. It is hence anticipated that research and development toward nanocellulose and composites will continue their exponential growth in the coming decades. Harnessed synergistically with biomedical paradigms, this abundant natural polymer offers great promise for sustainable use in biomedical technologies and applications.

## Conflict of Interest

The authors declare no conflict of interest.
